# An annotated checklist of free-living Copepoda (Crustacea) of Iraq: diversity, distribution, and biogeographical affinities

**DOI:** 10.3897/zookeys.1292.196683

**Published:** 2026-09-16

**Authors:** Murtada Naser, Amaal Yasser, Maciej Karpowicz, Patricio R. de Los Ríos-Escalante

**Affiliations:** 1 School of Environment and Science, Griffith University, 170 Kessels Road, Nathan, Queensland 4111, Australia School of Environment and Science, Griffith University Queensland Australia; 2 Biodiversity Research, University of Vienna, Rennweg 14, 1030 Vienna, Austria Biodiversity Research, University of Vienna Vienna Austria; 3 Department of Hydrobiology, Faculty of Biology, University of Bialystok, Bialystok, Poland Department of Hydrobiology, Faculty of Biology, University of Bialystok Bialystok Poland; 4 Departamento de Ciencias Biológicas y Químicas, Facultad de Recursos Naturales, Universidad Católica de Temuco, Temuco 4810399, Chile Departamento de Ciencias Biológicas y Químicas, Facultad de Recursos Naturales, Universidad Católica de Temuco Temuco Chile; 5 Núcleo de Estudios Ambientales, Facultad de Recursos Naturales, Universidad Católica de Temuco, Temuco 4810399, Chile Núcleo de Estudios Ambientales, Facultad de Recursos Naturales, Universidad Católica de Temuco Temuco Chile

**Keywords:** Arabian Gulf, biogeographic transition zone, Calanoida, Cyclopoida, estuarine ecosystems, Harpacticoida, Mesopotamian marshes, Tigris–Euphrates Basin

## Abstract

Copepods are a major component of aquatic zooplankton and play essential roles in ecosystem functioning, yet their diversity in Iraq has remained poorly documented and taxonomically fragmented. This study presents the first comprehensive checklist of free-living Copepoda in Iraq, based on published records spanning 1921–2025. Only free-living taxa of Calanoida, Cyclopoida, and Harpacticoida were included, excluding parasitic forms and unidentified larval stages. A total of 51 confirmed species were compiled, comprising 26 Calanoida, 20 Cyclopoida, and 5 Harpacticoida. These include 22 freshwater-associated taxa (43.1%) and 29 marine-origin taxa (56.9%), the latter largely associated with Arabian Gulf estuarine and coastal plankton communities. Calanoida represent the most diverse order, comprising both freshwater taxa from inland waters and numerous marine-origin taxa associated with southern Iraq, whereas Cyclopoida dominate inland freshwater rivers and marshes. Harpacticoida remain markedly underrepresented, indicating substantial undersampling of benthic and meiofaunal habitats. The fauna reflects the coexistence of freshwater taxa associated with the Tigris–Euphrates Basin and marine taxa associated with the northwestern Arabian Gulf. Notable records include the endemic *Phyllodiaptomus irakiensis* and the invasive estuarine cyclopoid *Limnoithona tetraspina*, highlighting both regional endemism and emerging anthropogenic pressures. This checklist establishes the first modern national baseline for Iraqi copepod biodiversity and demonstrates that actual diversity remains substantially underestimated, particularly in estuarine, coastal, groundwater, and benthic ecosystems.

## Introduction

Copepods are among the most abundant and ecologically important organisms in aquatic ecosystems globally, performing vital functions within food webs, nutrient cycling processes, and energy transfer between freshwater, brackish, and marine habitats (Boxshall and Defaye 2007; Boxshall et al. 2016). As primary consumers and key intermediates in aquatic food webs, they are often used as indicators of environmental change and water quality. Changes in their diversity, abundance, and community structure serve as indicators of ecosystem disturbance and pollution (Reid and Williamson 2010; Benedetti et al. 2025; Yusuf et al. 2025).

The diversity patterns of free-living copepods on a global scale are mainly shaped by three major orders Calanoida, Cyclopoida, and Harpacticoida, whose distribution and community composition are influenced by habitat type, salinity gradients, hydrological connectivity, habitat origin (natural versus artificial), habitat age, and historical biogeographical processes (Boxshall and Defaye 2007; Morales-Ramírez et al. 2014). Whereas cyclopoids and harpacticoids are most common in freshwater systems, calanoids often dominate estuarine and marine plankton communities (Corgosinho et al. 2019; Sanoamuang and Dabseepai 2021).

Iraq occupies a unique biogeographical position at the crossroads of the Western Palearctic, Oriental, and Afro-tropical biogeographical influences. Combined with pronounced climatic gradients and a diverse array of freshwater, estuarine, and marine habitats, this setting makes Iraq an important region for studying aquatic biodiversity. Its aquatic habitats form a continuous hydrological gradient extending from inland rivers and marshes to estuarine and coastal waters, creating opportunities for the coexistence of both Palearctic freshwater taxa and tropical marine-derived species (Al-Yamani et al. 2007; Naser et al. 2025).

Despite this ecological significance, knowledge of Iraqi copepods remains fragmented and incomplete. Existing records are scattered across isolated local studies on rivers, marshes, estuaries, and coastal waters (e.g., Abdulwahab and Rabee 2015; Jebir et al. 2021; Majeed et al. 2021; Al-Keriawy and Al-Kalidy 2023), often using inconsistent taxonomic frameworks and outdated nomenclature. A previous review by Merza (2017) compiled a checklist of zooplankton in Iraqi waters, reporting 185 taxa, including 83 copepod records; however, this work combined Cladocera and Copepoda, relied primarily on uncritical literature compilation, and did not apply modern taxonomic standardization or verification of species identities. Consequently, a unified, critically revised, and taxonomically consistent national checklist focused specifically on Copepoda has not been available for Iraq.

The present study addresses this gap by providing the first comprehensive, taxonomically standardized checklist of free-living copepods recorded from Iraq. This checklist includes all free-living copepods recorded from Iraqi waters, encompassing freshwater, estuarine, and marine habitats of the northwestern Arabian Gulf. By synthesizing historical and contemporary literature, updating nomenclature in line with current taxonomy, and classifying species based on ecological origin, the study also provides annotated species-level accounts, documenting distribution patterns and biogeographic and ecological affinities. Together, these elements aim to establish a robust baseline framework for future taxonomic, ecological, and biogeographical research on Iraqi copepod fauna.

## Materials and methods

This checklist results from a comprehensive review of the published literature on copepods reported from Iraq between 1921 and 2025. The review encompasses records from all Iraqi aquatic environments, including inland freshwater ecosystems, estuarine and transitional waters, and marine waters of the northwestern Arabian Gulf. These sources included peer-reviewed journal articles, regional journals, academic theses, monographs, and technical reports. Historical species records, including those from early faunal studies such as Gurney (1921), were included only when their taxonomic identities could be confirmed with high confidence. For each record, species name, locality, habitat, and bibliographic source were extracted and cross-checked to avoid duplication and ensure consistency.

Only free-living copepod species belonging to Calanoida, Cyclopoida, and Harpacticoida were included. Parasitic copepods, larval stages not identifiable to species, and doubtful or unresolved records were excluded.

Records were accepted only when: species-level identification was provided; locality was clearly occurring within Iraq; and nomenclature could be validated. Taxonomically doubtful records were omitted when identification was inconsistent with known regional biogeography (see Suppl. material 1 for the full list of excluded and doubtful taxa).

Species names, authorities, and taxonomic status were standardized according to the World Register of Marine Species (WoRMS), which served as the primary taxonomic reference. Synonyms, obsolete names, and misspellings in older literature were updated to the currently accepted nomenclature. Because this checklist is based on published records and no re-examination of voucher material was conducted, records reported at subspecies level were generally treated at the species level unless subspecific identification was explicitly supported by the original source or subsequent taxonomic revision. Taxonomic decisions and doubtful records were further evaluated using original descriptions and subsequent revisionary literature. Historical records reported under widely used generic names such as *Cyclops* and *Diaptomus* were revised where possible to reflect current taxonomy (see Table 1 and Suppl. material 1). Although these genera remain valid, their historical usage was considerably broader, and many taxa have since been reassigned to other genera following major taxonomic revisions over the past century.

**Table 1.** Checklist of free-living Copepoda recorded from Iraq: order Calanoida.

**Family**	**Species (accepted name)**	**Habitat**	**Locality in Iraq**	**Status**	**Reference**
Acartiidae	*Acartiella faoensis* Khalaf, 1991	Brackish estuarine	Khor Al-Zubair	Native	Khalaf (1991); Morad et al. (2013)
Acartiidae	Acartia (Odontacartia) ohtsukai Ueda & Bucklin, 2006	Brackish estuarine	Shatt Al-Arab	Native	Jebir et al. (2025)
Acartiidae	Acartia (Odontacartia) pacifica (Steuer, 1915)	Marine	Northwestern Arabian Gulf	Native	Morad et al. (2013)
Clausocalanidae	*Clausocalanus minor* Sewell, 1929	Marine-estuarine	Shatt Al-Arab estuary	Native	Jebir et al. (2025)
Diaptomidae	*Acanthodiaptomus denticornis* (Wierzejski, 1887)	Freshwater	Southern Iraqi marshes	Native	Mohamed and Salman (2009)
Diaptomidae	Arctodiaptomus (Rhabdodiaptomus) salinus (Daday, 1885)	Freshwater–brackish	Southern Iraqi marshes	Native	Mohamed and Salman (2009); Ajeel (2012); Al-Taee et al. (2018)
Diaptomidae	*Eudiaptomus vulgaris* (Schmeil, 1897)	Freshwater	Ezra’s Tomb marsh near Amara (Tigris)	Native	Gurney (1921)
Diaptomidae	*Metadiaptomus chevreuxi* (Guerne & Richard, 1894)	Freshwater	Amara region	Native	Gurney (1921)
Diaptomidae	Phyllodiaptomus (Phyllodiaptomus) blanci (Guerne & Richard, 1896)	Freshwater	Tigris floodplain near Amara	Native	Gurney (1921)
Diaptomidae	Phyllodiaptomus (Phyllodiaptomus) irakiensis Khalaf, 2008	Freshwater	Southern marshes, Shatt Al-Arab basin	Endemic	Khalaf (2008b); Mohamed and Salman (2009)
Eucalanidae	*Subeucalanus subcrassus* (Giesbrecht, 1888)	Marine	Northwestern Arabian Gulf	Native	Morad et al. (2013)
Euchaetidae	*Euchaeta concinna* Dana, 1849	Marine	Northwestern Arabian Gulf	Native	Morad et al. (2013)
Paracalanidae	*Bestiolina arabica* Ali, Al-Yamani & Prusova, 2007	Brackish estuarine	Shatt Al-Arab estuary	Native	Khalaf (2008a)
Paracalanidae	*Paracalanus parvus* (Claus, 1863)	Marine-estuarine	Shatt Al-Arab estuary	Native	Jebir et al. (2025)
Paracalanidae	*Acrocalanus gibber* Giesbrecht, 1888	Marine	Northwestern Arabian Gulf	Native	Ajeel (2012); Khalaf (1988); Morad et al. (2013)
Paracalanidae	*Paracalanus aculeatus* Giesbrecht, 1888	Marine	Northwestern Arabian Gulf	Native	Ajeel (2012); Khalaf (1988); Morad et al. (2013)
Paracalanidae	*Parvocalanus crassirostris* (Dahl, 1894)	Marine	Northwestern Arabian Gulf	Native	Ajeel (2012); Khalaf (1988); Morad et al. (2013)
Calanidae	*Canthocalanus pauper* (Giesbrecht, 1888)	Marine	Northwestern Arabian Gulf	Native	Khalaf (1988)
Centropagidae	*Centropages tenuiremis* Thompson I.C. & Scott A., 1903	Marine	Northwestern Arabian Gulf	Native	Khalaf (1988); Morad et al. (2013)
Pseudodiaptomidae	*Pseudodiaptomus ardjuna* Brehm, 1953	Brackish estuarine	Shatt Al-Basrah Canal	Native	Mohamed (2011)
Pseudodiaptomidae	*Pseudodiaptomus marinus* Sato, 1913	Marine	Northwestern Arabian Gulf	Probable non-native	Khalaf (1992); Morad et al. (2013)
Pontellidae	*Labidocera minuta* Giesbrecht, 1889	Marine	Northwestern Arabian Gulf	Native	Khalaf (1988)
Pontellidae	*Labidocera kroyeri stylifera* Thompson I.C. & Scott A., 1903	Marine	Northwestern Arabian Gulf	Native	Khalaf (1992)
Pontellidae	*Labidocera bengalensis* Krishnaswamy, 1952	Marine	Northwestern Arabian Gulf	Native	Khalaf (1992)
Temoridae	*Temora turbinata* (Dana, 1849)	Marine	Northwestern Arabian Gulf	Native	Khalaf (1988); Morad et al. (2013)
Tortanidae	Tortanus (Tortanus) forcipatus (Giesbrecht, 1889)	Marine	Northwestern Arabian Gulf	Native	Morad et al. (2013)

Species were classified into two major ecological groups based on ecological origin and biogeographical affinity:

1. Freshwater species – taxa associated primarily with inland continental waters, including rivers, lakes, reservoirs, canals, marshes, and inland brackish transitional habitats.
2. Marine-origin species – taxa derived from Arabian Gulf marine lineages occurring in estuarine and marine-influenced waters of southern Iraq.

This classification reflects biological affinity rather than rigid salinity thresholds and better captures the dual freshwater–marine structure of Iraqi copepod fauna.

## Results

**Figure 1.**
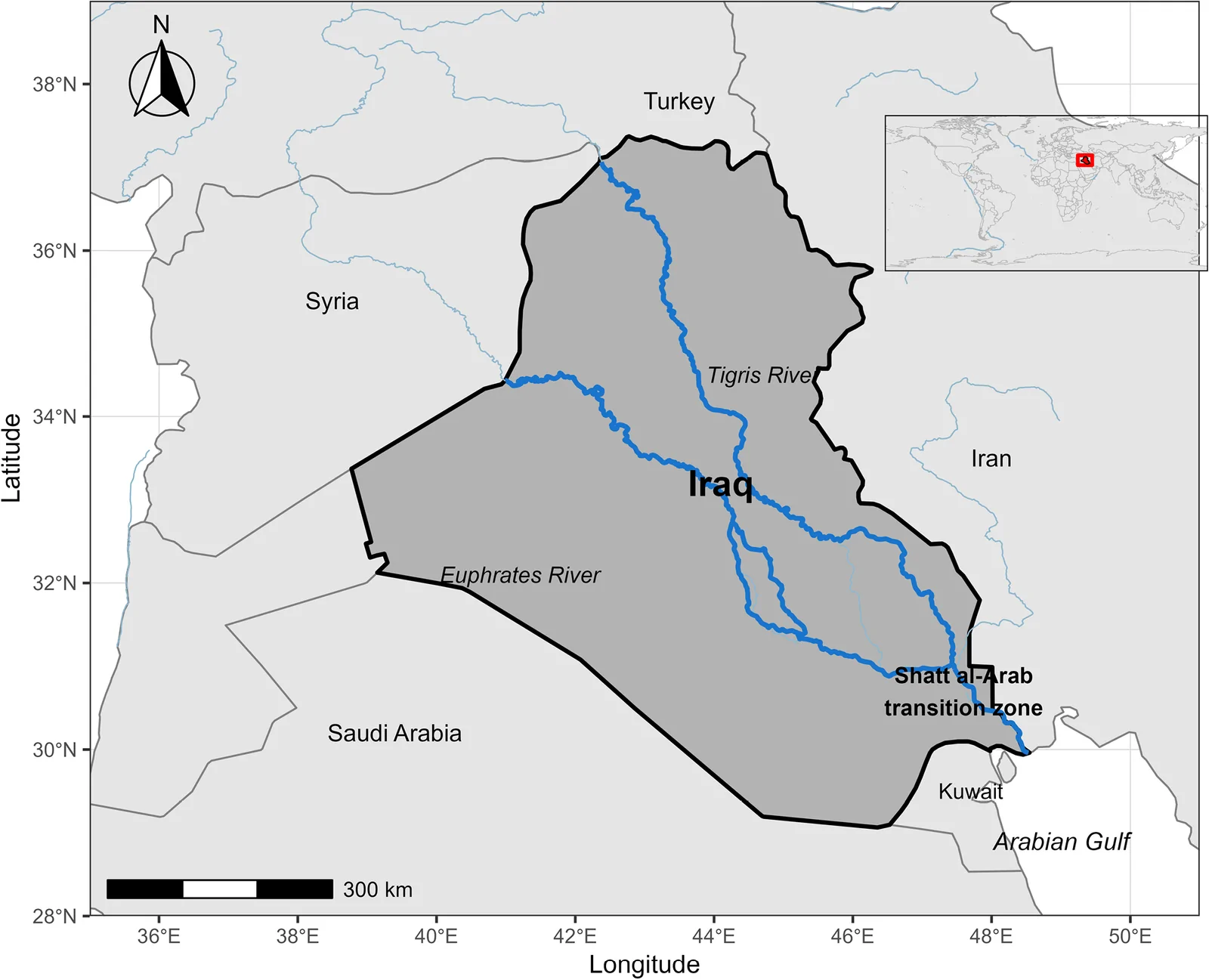
Regional geographic context of Iraq showing the Tigris–Euphrates river system, the Shatt al-Arab estuary, and the northwestern Arabian Gulf. The inset world map highlights the location of the study area within a global context.

The checklist of free-living copepods recorded from Iraq now comprises 51 species, belonging to three orders: Calanoida (26 species), Cyclopoida (20 species), and Harpacticoida (5 species). Cyclopoida remain the dominant group in freshwater fauna, which includes most of the records obtained from inland rivers, marshes, and reservoirs. On the other hand, Calanoida includes both their diaptomid freshwater genera as well as a large number of estuarine–marine taxa from Arabian Gulf plankton communities. Harpacticoida remain underrepresented in biodiversity surveys, most likely because benthic, littoral, and meiofaunal habitats have received less sampling effort than planktonic environments, rather than because of low species diversity. Of the 51 recorded copepod species currently known from Iraq, 22 species (43.1%) are associated with freshwater habitats, whereas 29 species (56.9%) are associated with marine and estuarine habitats connected to the northwestern Arabian Gulf. Freshwater species dominate inland habitats such as rivers, canals, marshes, lakes, and reservoirs, reflecting the strong continental character of the Tigris–Euphrates basin. In contrast, marine-derived species are concentrated mainly in southern Iraq, particularly in the Shatt al-Arab estuary, Shatt al-Basrah Canal, Khor al-Zubair, and adjacent northwestern Arabian Gulf waters, where tidal Gulf intrusion creates extensive brackish and marine transitional habitats (Fig. 1).

**Table 2.** Checklist of free-living Copepoda recorded from Iraq: order Cyclopoida.

**Family**	**Species (accepted name)**	**Habitat**	**Locality in Iraq**	**Status**	**Reference**
Corycaeidae	Corycaeus (Ditrichocorycaeus) dahli Tanaka, 1957	Marine	Northwestern Arabian Gulf	Native	Morad et al. (2013)
Corycaeidae	*Ditrichocorycaeus lubbocki* (Giesbrecht, 1891)	Marine	Northwestern Arabian Gulf	Native	Morad et al. (2013)
Corycaeidae	*Ditrichocorycaeus andrewsi* (Farran, 1911)	Marine	Northwestern Arabian Gulf	Native	Morad et al. (2013)
Cyclopidae	*Cyclops vicinus* Uljanin, 1875	Freshwater	Amara region wetlands, Tigris-Euphrates basin	Native	Gurney (1921); Muften et al. (2002); Al-Doori (2009)
Cyclopidae	*Diacyclops bicuspidatus* (Claus, 1857)	Freshwater	Tigris floodplain wetlands	Native	Gurney (1921)
Cyclopidae	*Diacyclops languidoides* (Lilljeborg, 1901)	Freshwater	Dukan Reservoir, Tigris basin	Native	Dhahir and Ali (2017)
Cyclopidae	*Eucyclops agilis agilis* (Koch, 1838)	Freshwater	Amara region wetlands, Tigris-Euphrates basin	Native	Gurney (1921); Al-Laami, (1998); Al-Sodani et al. (2007); Matlob (2004); Muften et al. (2002).
Cyclopidae	*Macrocyclops albidus* (Jurine, 1820)	Freshwater	Southern Iraqi marshes, Tigris-Euphrates basin	Native	Gurney (1921); Abdulwahab and Rabee (2015); Matlob (2004)
Cyclopidae	*Megacyclops viridis viridis* (Jurine, 1820)	Freshwater	Tigris River basin	Native	Gurney (1921)
Cyclopidae	*Mesocyclops leuckarti* (Claus, 1857)	Freshwater	Tigris River basin	Native	Gurney (1921)
Cyclopidae	*Microcyclops diaphanus* (Fischer, 1853)	Freshwater	Tigris	Native	Gurney, (1921)
Cyclopidae	*Apocyclops dimorphus* (Kiefer, 1934)	Freshwater	Tigris-Euphrates basin	Native	Al-Doori (2009, 2012)
Cyclopidae	*Apocyclops dengizicus dengizicus* (Lepeshkin, 1900)	Freshwater	Tigris-Euphrates basin, Garmat Ali, Shatt Al-arab	Native	Gurney, (1921); Mohamed et al. (2004, 2008)
Cyclopidae	*Paracyclops fimbriatus fimbriatus* (Fischer, 1853)	Freshwater	Habbaniya Lake	Native	Abdulwahab and Rabee (2015); Muften et al. (2002)
Cyclopidae	*Paracyclops affinis* (Sars G.O., 1863)	Freshwater	Southern Iraqi marshes	Native	Gurney (1921); Abdulwahab and Rabee (2015); Al-Doori (2009); Matlob (2004)
Cyclopidae	Microcyclops (Microcyclops) varicans (Sars G.O., 1863)	Freshwater	Euphrates basin (Karbala), Garma marshes	Native	Hussain (2019); Al-Saboonchi et al. (1986)
Cyclopidae	*Thermocyclops crassus* (Fischer, 1853)	Freshwater	Tigris River basin; Garma marshes	Native	Gurney (1921); Saboonchi et al. (1986)
Cyclopidae	*Ectocyclops phaleratus* (Koch, 1838)	Freshwater	Tigris River, Baghdad	Native	Majeed et al. (2021); Nashaat and Al-Bahathy (2022)
Oithonidae	*Limnoithona tetraspina* Zhang & Li, 1976	Brackish estuarine	Shatt Al-Arab	Probable non-native	Mohammed et al. (2014)
Oithonidae	*Oithona attenuata* Farran, 1913	Marine	Northwestern Arabian Gulf	Native	Morad et al. (2013)

**Table 3.** Checklist of free-living Copepoda recorded from Iraq: order Harpacticoida.

**Family**	**Species (accepted name)**	**Habitat**	**Locality in Iraq**	**Status**	**Reference**
Canthocamptidae	Canthocamptus (Canthocamptus) staphylinus staphylinus Jurine, 1820	Freshwater	Southern Iraqi marshes, Tigris basin	Native	Gurney (1921)
Miraciidae	*Macrosetella gracilis* (Dana, 1848)	Marine	Northwestern Arabian Gulf	Native	Morad et al. (2013)
Miraciidae	*Delavalia longifurca* (Sewell, 1934)	Brackish estuarine	Shatt Al-Arab and southern marshes	Native	Mohammed (2018)
Peltidiidae	*Clytemnestra scutellata* Dana, 1848	Marine	Northwestern Arabian Gulf	Native	Morad et al. (2013)
Tachidiidae	*Euterpina acutifrons* (Dana, 1848)	Marine	Northwestern Arabian Gulf	Native	Morad et al. (2013)

This distribution pattern reflects the coexistence of freshwater and marine-associated copepod assemblages in Iraq, corresponding to the country’s diverse aquatic habitats, which range from inland rivers, lakes, and marshes to estuarine and marine environments connected to the northwestern Arabian Gulf.

Distinct ecological differences are evident among the three copepod orders. Among Calanoida, marine taxa clearly outnumber freshwater and brackish taxa. Of the 26 recorded calanoid species, 5 are freshwater, 5 are brackish, and 16 are marine, indicating a substantial contribution of Arabian Gulf planktonic lineages to the currently documented calanoid fauna of Iraq. Cyclopoida, in contrast, are predominantly freshwater, with 15 of the 20 recorded species (75.0%) restricted to inland freshwater habitats. The remaining five species comprise primarily marine or brackish-water taxa, including estuarine and coastal oithonids occurring in southern Iraqi coastal waters. This distribution highlights the importance of cyclopoids as a major component of the continental freshwater copepod fauna of Iraq and contrasts with the stronger marine influence observed among calanoids.

Harpacticoida are now represented by five recorded species, including one freshwater species (*Canthocamptus staphylinus* Jurine, 1820), one estuarine species (*Delavalia longifurca*, Sewell, 1934), and three marine planktonic harpacticoids (*Macrosetella gracilis* Dana, 1848, *Clytemnestra scutellata* Dana, 1848, and *Euterpina acutifrons* Dana, 1848). Despite this increase, harpacticoid diversity in Iraq remains clearly underestimated, largely because benthic and meiofaunal habitats especially sediments, littoral substrates, and interstitial zones, have received very limited systematic sampling.

Several species in the Iraqi copepod fauna are of particular biogeographical and ecological significance. The only confirmed endemic copepod species documented in Iraq is the calanoid *Phyllodiaptomus irakiensis* Khalaf, 2008. This taxon was originally described from the Shatt al-Arab River in southern Iraq and can be regarded as a true Mesopotamian freshwater lineage. As such, it may represent a locally distinctive evolutionary lineage within Iraq’s inland-water fauna (Khalaf 2008b; Bekleyen et al. 2017). Due to its restricted distribution, it is a key taxon for conservation and phylogeographic research in the future.

Currently, the only known invasive species established in Iraq is the cyclopoid copepod *Limnoithona tetraspina* Zhang & Li, 1976. This estuarine copepod, originally native to East Asia, has established outside of its native range into the lower Shatt Al-Arab transitional zone via ballast water from maritime shipping (Mohammed et al. 2014; Gallardo et al. 2016). The invasive status of *L. tetraspina* is therefore of great concern as it has been shown to disturb estuarine plankton communities, reduce the diversity of native zooplankton and shift food web dynamics in brackish water ecosystems (Gallardo et al. 2016; Annabi-Trabelsi et al. 2019).

*Sinodiaptomus sarsi* Rylov, 1923, a species native to East Asia that has expanded into parts of Europe and western Asia, has also been reported from Iraq (Abdulwahab and Rabee 2015). However, because the Iraqi record requires taxonomic verification, the species is excluded from the accepted checklist pending confirmation. Consequently, the extent of non-native copepod establishment in Iraqi inland waters remains insufficiently understood. Future surveys integrating detailed morphological examination with molecular approaches will be essential to verify doubtful records and assess the potential spread of non-native copepods within the Tigris–Euphrates basin.

The annotated checklist (Tables 1–3) presents a taxonomically standardized summary of the relevant Iraqi copepod fauna with current nomenclature and dealt species status. Species are ordered into their sequences of order, family, and genus with information on the distribution, ecology and remarks for each species.

### Iraqi Calanoid copepods

#### Family Acartiidae Sars G.O., 1903

***Acartiella faoensis* Khalaf, 1991**

**Distribution**. Known from Khor Al-Zubair and Khor Abdulla, southern Iraq; later recorded from Kuwait, Iranian Gulf waters, and India (Khalaf 1991; Al-Yamani et al. 2009; Peyghan et al. 2011; Mukherjee et al. 2021).

**Remarks**. Originally described from Iraqi estuarine waters, this species is currently known primarily from the northwestern Arabian Gulf region, with southern Iraq representing the type locality and one of its best-documented areas of occurrence (Khalaf 1991; Al-Yamani et al. 2009). Subsequent records from Kuwait and Iran remain within the same Gulf biogeographic province, supporting its interpretation as a characteristic regional estuarine species of the northwestern Arabian Gulf. The known distribution of *Acartiella faoensis* suggests that the species may be largely restricted to Gulf and Gulf-associated estuarine environments, although its full geographic range remains incompletely understood. The Indian occurrence is biogeographically unusual and may reflect overlooked dispersal pathways or secondary introduction (Mukherjee et al. 2021). As one of the few copepod species originally described from Iraq, *A. faoensis* is of particular biogeographic interest and highlights the importance of the northwestern Arabian Gulf as a center of regional estuarine biodiversity.

##### Acartia (Odontacartia) ohtsukai Ueda & Bucklin, 2006

**Distribution**. Originally described from Ariake Bay, Japan, but subsequently recorded from the northwestern Arabian Gulf, including Gulf waters adjacent to Iraq and Kuwait (Al-Yamani et al. 2011).

**Remarks**. Although first recognized as an East Asian brackish-water species, *A. ohtsukai* is now confirmed from the Arabian Gulf, demonstrating a wider Indo-West Pacific distribution than initially understood. Its occurrence in the northwestern Arabian Gulf extends the known range of the species considerably westward from its original East Asian distribution and highlights the broad geographic connectivity of Indo–West Pacific estuarine copepod faunas. The record from Gulf waters adjacent to Iraq indicates that the species may occur in southern Iraqi marine or estuarine habitats, although confirmed Iraqi records remain limited. Its inclusion in the northwestern Arabian Gulf fauna indicates that occurrence in Iraqi marine or estuarine waters is biogeographically plausible rather than anomalous. However, because this species is morphologically close to *A. pacifica* and related Odontacartia taxa, accurate identification requires careful examination of female caudal ramus characters and male fifth-leg morphology. Iraqi records should still be supported by careful examination of female caudal ramus characters and male fifth-leg morphology, ideally complemented by molecular confirmation (Ueda and Bucklin 2006; Al-Yamani et al. 2011). Additional surveys in Iraqi coastal and estuarine waters will be necessary to clarify the local distribution and status of this species within the northwestern Arabian Gulf region.

##### Acartia (Odontacartia) pacifica (Steuer, 1915)

**Distribution**. Recorded from southern Iraqi marine waters in the northwestern Arabian Gulf, where Morad et al. (2013) documented the species among the copepod fauna of Iraqi coastal plankton assemblages. It is not included in the Arabian Gulf checklist of Al-Yamani et al. (2011), suggesting that its Gulf distribution remains incompletely documented or under-recorded in regional surveys.

**Remarks**. Acartia (Odontacartia) pacifica is a warm-water marine calanoid copepod of the family Acartiidae, widely distributed in tropical and subtropical Indo–West Pacific coastal waters. It belongs to the *A. erythraea* species group and is now recognized as part of a cryptic species complex that includes closely related taxa such as *A. ohtsukai* and *A. edentata* (Ueda and Bucklin 2006; Srinui et al. 2019). The species is typically associated with high-salinity coastal marine waters rather than brackish estuaries, and it is ecologically important as an omnivorous plankton feeder and producer of long-lived resting eggs that form sediment egg banks (Jiang et al. 2004). The Iraqi record is noteworthy because species belonging to the *A. erythraea* complex are frequently difficult to distinguish using routine morphological examinations, and historical records may require re-evaluation in light of modern taxonomic revisions. Given the absence of confirmed records in major regional syntheses of Arabian Gulf copepods, the occurrence of *A. pacifica* in Iraqi waters should be regarded as requiring additional verification through detailed morphological and, ideally, molecular analyses. Future surveys will be important for determining whether the Iraqi material truly represents *A. pacifica* or another closely related member of the Odontacartia complex.

#### Family Centropagidae Giesbrecht, 1893

##### *Centropages tenuiremis* Thompson & Scott, 1903

**Distribution**. Recorded from southern Iraqi marine waters in the northwestern Arabian Gulf, where Morad et al. (2013) documented it among the copepod fauna of Iraqi coastal plankton assemblages. The species is also listed in Al-Yamani et al. (2011), confirming its established occurrence in Arabian Gulf plankton communities.

**Remarks**. *Centropages tenuiremis* is a warm-water marine calanoid copepod of the family Centropagidae, widely distributed in tropical and subtropical coastal waters of the Indo-West Pacific, including the Arabian Gulf, India, Southeast Asia, and East Asia (Zhang et al. 2011; Hamdan and Rahim 2023). It is a characteristic neritic species often seasonally dominant in coastal plankton and is recognized for its ecological flexibility, including omnivorous feeding and dual egg production strategies (subitaneous and diapause eggs), which enhance survival in fluctuating environments (Wu et al. 2007; Wu et al. 2008). The species is frequently associated with productive coastal and estuarine ecosystems, where it may become a dominant component of mesozooplankton communities during favorable environmental conditions. Its occurrence in Iraqi marine waters is consistent with its broad distribution throughout the Arabian Gulf and wider Indo–West Pacific region. The records from Iraq and neighboring Gulf waters indicate that *C. tenuiremis* forms part of the characteristic neritic copepod assemblage of the northwestern Arabian Gulf. The ability to produce both subitaneous and diapause eggs likely contributes to its persistence in the highly variable environmental conditions that characterize shallow Gulf ecosystems.

#### Family Clausocalanidae Giesbrecht, 1893

##### *Clausocalanus minor* Sewell, 1929

**Distribution**. Recorded from Iraqi estuarine waters influenced by Arabian Gulf marine intrusion (Jebir et al. 2025) and also listed in the northwestern Arabian Gulf zooplankton fauna by Al-Yamani et al. (2011), confirming its established occurrence in Gulf waters adjoining southern Iraq.

**Remarks**. *Clausocalanus minor* is a marine planktonic calanoid typical of tropical and subtropical shelf and coastal waters, widely distributed in Indo-West Pacific marine systems (Bellardini et al. 2024; Kim et al. 2025). The species is commonly associated with coastal and shelf plankton communities, where it contributes to the small-calanoid component of marine mesozooplankton assemblages. Its occurrence in Iraqi estuarine waters is consistent with its known distribution throughout the Arabian Gulf and adjacent Indo–West Pacific marine regions. The records from southern Iraq and neighboring Gulf waters indicate that *C. minor* forms part of the characteristic plankton fauna of the northwestern Arabian Gulf. As a small planktonic copepod, it contributes to secondary production and serves as an important trophic link between phytoplankton and higher consumers within Gulf pelagic food webs.

#### Family Diaptomidae Baird, 1850

##### *Acanthodiaptomus denticornis* (Wierzejski, 1887)

**Distribution**. Al-Huwaizah Marsh and Al-Hammar Marsh, southern Iraq (Mohamed and Salman 2009).

**Remarks**. Reported by Mohamed and Salman (2009) based on morphological identification and accompanying illustrations. Although the published figures appear compatible with *Acanthodiaptomus* and plausibly with *A. denticornis*, the occurrence of this predominantly temperate species in the lowland Mesopotamian marshes is biogeographically and ecologically unexpected. Consequently, this record should be regarded as provisional pending confirmation through new collections and integrated morphological and molecular analyses. The Iraqi populations may represent a cryptic species or a locally adapted lineage closely related to *A. denticornis*.

##### Arctodiaptomus (Rhabdodiaptomus) salinus (Daday, 1885)

**Distribution**. Recorded from southern Iraqi marshes and inland freshwater–brackish transitional wetlands (Mohamed and Salman 2009).

**Remarks**. This euryhaline diaptomid is widely distributed in inland saline lakes and subsaline wetlands across the western Palearctic and Mediterranean region (Marrone et al. 2014, 2017). Its Iraqi occurrence is consistent with its known tolerance of elevated salinity and reflects the natural salinity gradients of Mesopotamian marsh ecosystems, where freshwater and subsaline habitats intermix (Mohamed and Salman 2009; Naser et al. 2025). The species is widely regarded as one of the characteristic copepods of inland saline and brackish continental waters and is often associated with habitats subject to marked seasonal fluctuations in salinity. Its occurrence in southern Iraq highlights the ecological importance of salinity as a structuring factor within Mesopotamian wetlands and demonstrates the coexistence of both freshwater and halophilic copepod elements within the region. Biogeographically, the Iraqi populations represent part of a broad western Palearctic saline-water fauna extending from the Mediterranean Basin through the Middle East into western Asia.

##### *Eudiaptomus vulgaris* (Schmeil, 1897)

**Distribution**. Recorded from the permanent marshes of Ezra’s Tomb near Amara in southern Iraq, where it was reported as common by Gurney (1921), and more recently from the Al-Hussainiya River in Karbala Province (Kadhim 2025).

**Remarks**. *Eudiaptomus vulgaris* is a freshwater calanoid copepod widely distributed in Europe and western Asia. Comparative studies indicate that the species is morphologically distinct from the closely related *Eudiaptomus transylvanicus* and occupies a variety of freshwater habitats, including floodplain wetlands, rivers, ponds, and lakes (Podshivalina et al. 2022). The occurrence of *E. vulgaris* in Iraq is biogeographically noteworthy because the species is generally associated with temperate Palearctic freshwater faunas. Gurney (1921) specifically highlighted its occurrence in Mesopotamia as a significant extension of its known range. The recent record from the Al-Hussainiya River confirms that the species persists in Iraqi inland waters and is not solely known from historical collections (Kadhim 2025). Its presence contributes to the distinctive Palearctic element of the Iraqi copepod fauna.

##### *Metadiaptomus chevreuxi* (Guerne & Richard, 1894)

**Distribution**. Recorded from inland freshwater habitats of Iraq, including temporary ponds and wetlands of the Tigris–Euphrates basin, where it represents one of the characteristic calanoid taxa of ephemeral inland waters (Gurney 1921; Rayner 2000; Marrone et al. 2020).

**Remarks**. *Metadiaptomus chevreuxi* is a freshwater calanoid copepod of the family Diaptomidae (subfamily Paradiaptominae), representing one species of the subfamily Paradiaptominae occurring outside Africa (Rayner 2000; Marrone and Naselli-Flores 2005; Marrone et al. 2020). It is a morphologically stable species with well-defined diagnostic characters, although minor geographic variation in furcal setation has been documented among Mediterranean and Middle Eastern populations (Jaume 1989; Marrone and Naselli-Flores 2005). Biogeographically, it is considered a North African–Middle Eastern arid-zone species whose range extends eastward into Iraq and Iran (Rayner 2000). Its known distribution forms three disjunct regional clusters: the western Mediterranean (Maghreb, mainland Spain, Mallorca, Sicily), central Saharan oases, and the Middle East (Jordan, Iraq, Iran), reflecting a fragmented western Palearctic arid-belt distribution (Marrone et al. 2020). Ecologically, the species is strongly associated with small, isolated temporary ponds and ephemeral freshwater pools in semi-arid and arid landscapes, where it is a characteristic element of desert and steppe crustacean communities (Jaume 1989). The occurrence of this species in Iraq is biogeographically significant because it represents one of the easternmost confirmed populations of a predominantly North African–Middle Eastern arid-zone lineage. Its presence highlights the affinity of Mesopotamian temporary waters with other arid-region freshwater ecosystems across the western Palearctic and contributes to the distinctive biogeographical character of the Iraqi freshwater copepod fauna. Iraqi populations are therefore of high biogeographic significance, representing the easternmost extension of this predominantly North African arid-zone lineage and reinforcing faunal links between Mesopotamian temporary waters and western Palearctic desert freshwater systems.

##### Phyllodiaptomus (Phyllodiaptomus) blanci (Guerne & Richard, 1896)

**Distribution**. Recorded from freshwater habitats in the Amara region of southern Iraq based on the historical record of Gurney (1921). Outside Iraq, the species is widely distributed from Central Asia through China and Southeast Asia, with westward extensions into the Middle East, including Israel and the northwestern Arabian Gulf region (Dumont and Ranga Reddy 1993; Dumont et al. 1996; Al-Yamani et al. 2011; Marrone et al. 2014).

**Remarks**. *Phyllodiaptomus blanci* is the type species of the genus *Phyllodiaptomus* and defines the blanci-group within the nominate subgenus Phyllodiaptomus (Phyllodiaptomus) (Dumont and Ranga Reddy 1993; Dumont et al. 1996). This group is characterized by distinctive male right fifth legs with modified second exopodal segments and females with reduced or modified lateral wings of the fifth pediger and characteristic genital somite morphology (Dumont et al. 1996; Sanoamuang and Yindee 2001; Teeramaethee and Sanoamuang 2006). The historical Iraqi record reported by Gurney (1921) confirms the presence of this Asian diaptomid lineage in Mesopotamian freshwaters. Its wider regional distribution and the native status of populations reported outside the Tigris–Euphrates Basin require further assessment. Because *P. blanci* serves as the principal reference species for all blanci-group comparisons, it remains taxonomically central for evaluating Iraqi *Phyllodiaptomus*, especially *P. irakiensis*. The occurrence of *P. blanci* in Iraq, together with records from the Middle East and northwestern Arabian Gulf region, suggests historical biogeographic connectivity among western Asian freshwater systems and supports the hypothesis that Iraqi *Phyllodiaptomus* populations form part of a broader Middle Eastern dispersal corridor.

##### Phyllodiaptomus (Phyllodiaptomus) irakiensis Khalaf, 2008

**Distribution**. Endemic to southern Iraq; known only from the Shatt Al-Arab River system, where it was originally described from freshwater low-salinity river habitats influenced by estuarine conditions (Khalaf 2008b).

**Remarks**. *Phyllodiaptomus irakiensis* is a well-defined endemic Iraqi species belonging to the blanci group of the nominate subgenus Phyllodiaptomus (Phyllodiaptomus) (Dumont and Reddy 1993; Dumont et al. 1996). It was described from the Shatt Al-Arab River and represents the second known species of *Phyllodiaptomus* recorded from Iraq (Khalaf 2008b). Although closely related to *P. blanci*, it differs by several stable diagnostic characters, including the conspicuous left metasomal wing expansion in females, dorsal hyaline lobes on the second and third urosomal somites, fine marginal furcal setulation, and structurally distinct male and female fifth legs (Khalaf 2008b). In males, the anal somite and furcal rami are characteristically curved to the right relative to the urosomal axis, and the right antennule differs from that of *P. blanci* (Khalaf 2008b). The occurrence of both *P. irakiensis* and the historically recorded *P. blanci* confirms the presence of the genus in the Tigris–Euphrates Basin and contributes to our understanding of its distribution in western Asia. This pattern supports the view that Mesopotamian freshwaters constitute an important biogeographic transition zone linking Middle Eastern aquatic ecosystems with the broader Central and Southeast Asian distribution of *Phyllodiaptomus*. Biogeographically, *P. irakiensis* is highly significant because it places Iraq at the westernmost confirmed limit of the genus’ main Asian distribution, linking Mesopotamian inland waters to the broader Central and Southeast Asian radiation of blanci group of *Phyllodiaptomus* species (Marrone et al. 2014; Bekleyen et al. 2017). As an endemic species currently known only from southern Iraq, *P. irakiensis* may represent a localized evolutionary derivative of an ancestral western Asian *Phyllodiaptomus* lineage. Its restricted distribution further emphasizes the conservation importance of the Shatt Al-Arab system and associated low-salinity freshwater habitats. Its presence contributes to documenting the regional distribution of this predominantly Asian calanoid lineage in southern Iraq.

#### Family Eucalanidae Giesbrecht, 1893

##### *Subeucalanus subcrassus* (Giesbrecht, 1888)

**Distribution**. Recorded from southern Iraqi marine waters in the northwestern Arabian Gulf, where Morad et al. (2013) listed it among the copepod fauna of Iraqi coastal plankton assemblages. The species is also included in the Arabian Gulf regional zooplankton checklist of Al-Yamani et al. (2011), confirming its established occurrence in Gulf marine waters adjacent to Iraq.

**Remarks**. *Subeucalanus subcrassus* is a warm-water marine calanoid copepod of the family Eucalanidae, widely distributed in tropical and subtropical neritic and oceanic waters. It is distinguished from congeners by the broad frontal head region, where head width exceeds height (Shih et al. 2022). This species is a characteristic component of warm high-salinity plankton communities and is often abundant in tropical shelf ecosystems, where it contributes significantly to copepod biomass and carbon transfer in marine food webs (Goetze and Ohman 2010; Beltrán-Castro et al. 2020). Within the Arabian Gulf, *S. subcrassus* is typically associated with warm, saline coastal and offshore waters and forms part of the characteristic Indo–West Pacific zooplankton assemblage that extends into the northwestern Gulf. In Iraqi waters, its presence in both Morad et al. (2013) and Al-Yamani et al. (2011) indicates that it is a native Arabian Gulf marine species naturally occurring in Iraq’s offshore and coastal plankton fauna rather than an occasional transient species. Its occurrence contributes to the marine component of the Iraqi copepod fauna and reflects the strong biogeographic affinity of the northwestern Arabian Gulf with tropical Indo–West Pacific marine ecosystems.

#### Family Euchaetidae Giesbrecht, 1893

##### *Euchaeta concinna* Dana, 1849

**Distribution**. Recorded from southern Iraqi marine waters in the northwestern Arabian Gulf, where Morad et al. (2013) documented the species among the copepod fauna of Iraqi coastal plankton assemblages. It is also included in the Arabian Gulf regional checklist of Al-Yamani et al. (2011), confirming that it is an established component of Gulf marine plankton communities.

**Remarks**. *Euchaeta concinna* (often treated as *Paraeuchaeta concinna*) is a large predatory marine calanoid copepod of the family Euchaetidae and belongs to the concinna species group recognized in the global revision of Euchaetidae (Park 1993). It is widely distributed in warm Indo-Pacific marginal seas, where it is characteristic of subtropical shelf and boundary-current waters (Jeong et al. 2011). Unlike small herbivorous paracalanids, this species is carnivorous and feeds mainly on smaller copepods, including genera such as *Acrocalanus*, *Paracalanus*, and *Parvocalanus*, thereby regulating lower trophic zooplankton populations (Wong et al. 2012). Within the Arabian Gulf, *E. concinna* forms part of the characteristic warm-water oceanic and shelf zooplankton assemblage and occupies a relatively high trophic position among copepods owing to its predatory feeding behavior. In Iraq, its occurrence in both Morad et al. (2013) and Al-Yamani et al. (2011) indicates that it is a native Arabian Gulf marine species naturally entering Iraqi coastal waters as part of offshore warm-water plankton assemblages. Its presence in Iraqi marine waters reflects the strong ecological and biogeographic connectivity between the northwestern Arabian Gulf and the broader Indo–West Pacific marine realm.

#### Family Paracalanidae Giesbrecht, 1893

##### *Bestiolina arabica* Ali, Al-Yamani & Prusova, 2007

**Distribution**. Recorded from Khor Al-Zubair canal and the Shatt Al-Arab River in southern Iraq (Khalaf 2008a) and also listed as part of the northwestern Arabian Gulf copepod fauna in Al-Yamani et al. (2011), confirming its broader Gulf distribution in waters adjoining Iraq and Kuwait.

**Remarks**. *Bestiolina arabica* is a native northwestern Arabian Gulf estuarine calanoid originally described from Kuwaiti coastal waters and recognized as a characteristic component of tropical Gulf plankton communities (Ali et al. 2007; Al-Yamani et al. 2007). Its confirmed occurrence in Iraqi estuarine waters and inclusion in the northwestern Arabian Gulf regional zooplankton fauna (Al-Yamani et al. 2011) demonstrate that it is a natural indigenous element of the Gulf–Shatt Al-Arab estuarine continuum rather than an introduced species. The species is particularly associated with warm, productive coastal and estuarine environments of the northwestern Arabian Gulf, where it contributes to the characteristic small-bodied calanoid assemblage of transitional waters. In Iraq, its presence reflects the natural hydrological and ecological connectivity between the Arabian Gulf and southern Iraqi estuaries, especially in Khor Al-Zubair and lower Shatt Al-Arab systems (Khalaf 2008a; Liu et al. 2021). Because *B. arabica* was originally described from the northwestern Arabian Gulf, its occurrence in Iraq is consistent with its known regional distribution and supports the recognition of the Gulf as a distinct biogeographic province for tropical and subtropical estuarine copepods. This species is therefore best interpreted as a native Gulf estuarine plankton taxon with stable regional distribution centered in the northwestern Arabian Gulf biogeographic province.

##### *Paracalanus parvus* (Claus, 1863)

**Distribution**. Recorded from estuarine waters of southern Iraq, including the Shatt Al-Arab and Shatt Al-Basrah estuarine systems (Jebir et al. 2025). The species is also included in the northwestern Arabian Gulf zooplankton fauna documented by Al-Yamani et al. (2011), confirming its regular occurrence in Gulf waters adjacent to Iraq and supporting its status as a natural Gulf-derived estuarine plankton taxon.

**Remarks**. *Paracalanus parvus* is a small calanoid copepod of the family Paracalanidae widely distributed in coastal and estuarine waters worldwide and traditionally regarded as a common native component of marine-estuarine zooplankton assemblages (Gomes et al. 2004; Miloslavić and Lučić 2015; Al-Hanoun and Mayya 2020). Its confirmed occurrence in both Iraqi estuaries and the broader northwestern Arabian Gulf fauna demonstrates that it is a regular native component of Gulf plankton communities rather than an introduced species. However, recent integrative taxonomic studies have shown that *P. parvus* represents a complex of multiple cryptic species, with molecular analyses recognizing at least 10–12 distinct taxa globally (Ueda et al. 2022). In several regions, populations formerly assigned to *P. parvus* have been reassigned to sibling species such as *P. indicus*, *P. tropicus*, and *P. orientalis* (Ueda et al. 2022). Consequently, Iraqi records identified as *P. parvus* should be treated provisionally until confirmed using modern morphological and molecular criteria. Ecologically, the species complex is characteristic of coastal and estuarine plankton, often dominant in nearshore food webs and adapted to marine-estuarine circulation patterns, including vertical migration behavior that facilitates persistence in fluctuating estuarine environments (Gomes et al. 2004; Jagadeesan et al. 2009). In Iraq, it is best interpreted as a native Gulf marine-estuarine taxon entering transitional waters through regular plankton exchange between the Arabian Gulf and lower Iraqi estuaries. The occurrence of *P. parvus parvus* in southern Iraq is consistent with its broad distribution throughout tropical and subtropical coastal waters of the Indo–West Pacific and adjacent seas. Although currently treated as a native component of the Iraqi estuarine fauna, future taxonomic studies may reveal that Gulf populations belong to one or more cryptic species within the *P. parvus* complex. Consequently, Iraqi records provide an important baseline for future integrative taxonomic investigations of Arabian Gulf paracalanids.

##### *Acrocalanus gibber* Giesbrecht, 1888

**Distribution**. Recorded from southern Iraqi marine–estuarine waters, including the Shatt Al-Arab River, Khor Al-Zubair, and adjacent northwestern Arabian Gulf-connected waters. In the Shatt Al-Arab, *A. gibber* has been documented as a dominant zooplankton component in middle and southern reaches across multiple sampling months, particularly in saline estuarine sectors influenced by Gulf intrusion (Maytham et al. 2019; Jebir et al. 2021). It is also recorded from Iraqi marine waters in the northwestern Arabian Gulf by Morad et al. (2013), who listed it among the characteristic calanoid copepods of southern Iraqi coastal plankton assemblages. Additional occurrences are known from Khor Al-Zubair and nearby Gulf-influenced channels as part of the warm-water marine plankton fauna (Khalaf 2008b; Ajeel 2012).

**Remarks**. *Acrocalanus gibber* is a small calanoid copepod of the family Paracalanidae, characteristic of warm tropical and subtropical coastal plankton communities. It is widely distributed in Indo–West Pacific neritic and shelf waters, where it frequently ranks among dominant small copepods in coastal mesozooplankton assemblages (Rakhesh et al. 2006; Lan et al. 2009; Long et al. 2021; Patnaik et al. 2025). In Iraqi waters, its occurrence across salinity ranges from mesohaline estuarine conditions (4–15‰) to nearly full marine salinity demonstrates broad tolerance to estuarine–marine gradients and confirms its adaptation to transitional Gulf–river systems (Maytham et al. 2019; Jebir et al. 2021).

The inclusion of *A. gibber* in Morad et al. (2013) confirms that the species is not confined to estuarine intrusion zones alone but is also a stable component of Iraq’s true marine coastal plankton fauna. Its occurrence in both estuarine and marine habitats is consistent with its ecological characteristics as a euryhaline coastal species widely distributed throughout tropical and subtropical Indo–West Pacific waters. In the northwestern Arabian Gulf, *A. gibber* represents a characteristic component of warm-water plankton assemblages and contributes to the regional marine biodiversity of Iraqi coastal waters. Ecologically, it functions as an herbivorous suspension feeder and an important grazer of phytoplankton, often contributing substantially to copepod biomass and trophic transfer to larval fish and higher consumers (He et al. 2023).

##### *Paracalanus aculeatus* Giesbrecht, 1888

**Distribution**. Recorded from southern Iraqi marine waters in the northwestern Arabian Gulf, where Morad et al. (2013) documented it among the characteristic marine copepods of Iraqi coastal plankton assemblages. It is also known from Kuwaiti Arabian Gulf waters, confirming a broader northwestern Gulf distribution (Michel et al. 1986).

**Remarks**. *Paracalanus aculeatus* is a warm-water marine calanoid copepod of the family Paracalanidae, widely distributed in tropical and subtropical coastal seas. It belongs to the distinct *P. aculeatus* group within *Paracalanus*, separate from the *P. parvus* complex (Bhattacharyya et al. 2023). The species is typical of saline neritic and shelf waters, where it often becomes abundant in coastal plankton communities and serves as an indicator of warm marine water masses (Das 2017; Long et al. 2021). Within the Arabian Gulf, *P. aculeatus* is a characteristic component of tropical marine zooplankton assemblages associated with coastal and shelf environments. Its occurrence in Iraqi waters is consistent with its broad distribution throughout warm Indo–West Pacific marine ecosystems and confirms its status as a native component of the northwestern Arabian Gulf plankton fauna. Ecologically, the species contributes to secondary production and energy transfer within marine food webs, forming part of the dominant small-calanoid community that supports larval fishes and other planktivorous organisms.

##### *Parvocalanus crassirostris* (Dahl, 1894)

**Distribution**. Recorded from southern Iraqi marine waters, where Morad et al. (2013) documented it among the copepod fauna of Iraqi coastal plankton communities. The species is also listed in the northwestern Arabian Gulf regional checklist of Al-Yamani et al. (2011), confirming that it is an established component of Arabian Gulf plankton assemblages.

**Remarks**. *Parvocalanus crassirostris* is a small warm-water calanoid copepod of the family Paracalanidae, common in tropical and subtropical coastal, estuarine, and shelf waters worldwide. It is frequently among the dominant small copepods in saline coastal ecosystems and is known for broad tolerance to estuarine salinity and temperature gradients (Tseng et al. 2008; Duggan et al. 2008). The species is morphologically variable, but molecular studies confirm that these morphotypes belong to a single genetically coherent species (Fattahi et al. 2025). Ecologically, it is an important herbivorous suspension feeder grazing mainly on nano- and microphytoplankton, and it plays a major trophic role linking phytoplankton production to larval fish and higher consumers (McKinnon et al. 2003; He et al. 2023). The occurrence of *Parvocalanus crassirostris* in southern Iraqi marine waters is consistent with its broad distribution in tropical and subtropical coastal ecosystems worldwide. Its presence in the northwestern Arabian Gulf reflects the warm, saline environmental conditions characteristic of the region. As a common small calanoid copepod, it likely contributes substantially to secondary production and energy transfer within Gulf pelagic food webs.

#### Family Calanidae Dana, 1846

##### *Canthocalanus pauper* (Giesbrecht, 1888)

**Distribution**. Recorded from Iraqi marine waters of the northwestern Arabian Gulf. The species was reported by Khalaf (1988) from the Iraqi sector of the Gulf and was subsequently included in regional zooplankton inventories of the Arabian Gulf by Al-Yamani et al. (2011), supporting its occurrence in the marine plankton communities of the region.

**Remarks**. *Canthocalanus pauper* is a cosmopolitan tropical and subtropical oceanic calanoid copepod widely distributed in warm marine waters of the Atlantic, Indian, and Pacific oceans. The species is typically associated with offshore and shelf-edge environments characterized by relatively high temperatures and salinities and is often regarded as an indicator of warm-water oceanic masses. It has been reported as a common component of zooplankton assemblages in tropical and subtropical seas, including regions influenced by major warm currents such as the Kuroshio and Taiwan Warm Current (Long et al. 2021; Shin et al. 2022). Recent studies have also identified the species as one of the dominant calanoid copepods in the Gulf of California, where its abundance is strongly associated with temperature-driven oceanographic processes (Rocha-Díaz et al. 2025). Its occurrence in the northwestern Arabian Gulf is consistent with its known ecological preferences for warm, saline marine environments and does not represent an unusual biogeographical record. In Iraq, *C. pauper* appears to be restricted to marine waters of the Arabian Gulf and has not been reported from estuarine or inland freshwater habitats (Khalaf 1988; Al-Yamani et al. 2011).

#### Family Centropagidae Giesbrecht, 1893

##### *Centropages tenuiremis* Thompson & Scott, 1903

**Distribution**. Recorded from Iraqi marine waters of the northwestern Arabian Gulf, including Khour Abdullah and adjacent Gulf waters, where it forms part of the coastal marine zooplankton assemblage (Khalaf 1988; Morad et al. 2013).

**Remarks**. *Centropages tenuiremis* is a warm-water coastal calanoid copepod widely distributed throughout the Indo-West Pacific region, including the Arabian Gulf, Arabian Sea, Indian Ocean, East China Sea, Taiwan Strait, and the western Pacific (Hamdan and Rahim 2023; Parthasarathi et al. 2024). The species is typically associated with coastal and shelf waters and is particularly characteristic of warm, disturbed, and estuarine-influenced marine environments (Xu et al. 2020). Studies from Southeast Asia have shown that *C. tenuiremis* can successfully colonize highly dynamic coastal ecosystems and may exhibit considerable ecological plasticity in response to fluctuating environmental conditions (Chew and Chong 2016; Xu et al. 2020). Its occurrence in the northwestern Arabian Gulf is therefore fully consistent with its known ecological preferences and broad Indo-West Pacific distribution and does not represent an unusual biogeographical record. In Iraq, the species appears restricted to marine waters of the Arabian Gulf and has not been reported from inland freshwater habitats (Khalaf 1988).

#### Family Pseudodiaptomidae Sars G.O., 1902

##### *Pseudodiaptomus ardjuna* (Brehm, 1953)

**Distribution**. Recorded from the Shatt Al-Arab River and adjacent brackish waters of southern Iraq (Mohamed 2011).

**Remarks**. This species is a marine-derived estuarine pseudodiaptomid widely distributed in tropical Indo-West Pacific brackish waters (Walter 1987). The species is typically associated with estuaries, coastal lagoons, and other transitional habitats where it tolerates substantial fluctuations in salinity. Its occurrence in the Shatt Al-Arab River is consistent with the estuarine character of the lower basin and with the known ecological preferences of the species. The Iraqi record represents part of the western distributional range of *P. ardjuna* within the northwestern Arabian Gulf region and contributes to our understanding of estuarine copepod diversity in southern Mesopotamia.

##### *Pseudodiaptomus marinus* Sato, 1913

**Distribution**. Recorded from southern Iraqi marine waters in the northwestern Arabian Gulf, where Morad et al. (2013) documented the species among the copepod fauna of Iraqi coastal plankton assemblages. At present, there is no confirmed record of this species in the Arabian Gulf checklist of Al-Yamani et al. (2011), indicating that its Gulf distribution may still be incompletely documented.

**Remarks**. *Pseudodiaptomus marinus* is a small estuarine–coastal calanoid copepod of the family Pseudodiaptomidae, originally native to the northwestern Pacific but now widely recognized as a globally invasive non-indigenous species in coastal and estuarine waters (Brylinski et al. 2012; Uttieri et al. 2020; Mohamad et al. 2024). It belongs to the *Pseudodiaptomus ramosus* group, composed mainly of marine and estuarine taxa (Shalaeva 2022). The species is highly tolerant of broad salinity gradients and is commonly dispersed via ballast water, ship traffic, and aquaculture transport.

The occurrence of *P. marinus* in Iraqi coastal waters is noteworthy because the species has expanded its range extensively beyond its native northwestern Pacific distribution through human-mediated transport. Although currently recorded from Iraqi marine waters, its broader distribution within the Arabian Gulf remains poorly documented. The apparent absence of confirmed records from the regional checklist of Al-Yamani et al. (2011) may indicate either incomplete regional sampling or a relatively recent arrival in Gulf waters. Consequently, the Iraqi record should be regarded as requiring further regional verification. Pending additional records from neighboring Gulf countries and molecular confirmation of Gulf populations, *P. marinus* is best considered a probable non-native species in Iraqi waters.

#### Family Pontellidae Dana, 1852–1853

##### *Labidocera minuta* Giesbrecht, 1889

**Distribution**. Recorded from southern Iraqi marine waters in the northwestern Arabian Gulf, where Morad et al. (2013) documented the species among the copepod fauna of Iraqi coastal plankton assemblages. It is also included in Al-Yamani et al. (2011), confirming its presence in Arabian Gulf plankton communities.

**Remarks**. *Labidocera minuta* is a warm-water neustonic calanoid copepod of the family Pontellidae, characteristic of tropical and subtropical Indo–West Pacific surface waters. It belongs to the *L. minuta* species group, distinguished by elongated female genital somites and slender fifth legs with reduced endopods (Murniati and Mulyadi 2018; Mulyadi 2020). The species is typically associated with near-surface marine plankton in warm coastal seas and is widely distributed from the Red Sea and Arabian Gulf to Southeast Asia and Australia (Johan et al. 2013; Abo-Taleb 2019). Its occurrence in Iraqi marine waters is consistent with its broad distribution throughout tropical and subtropical Indo–West Pacific ecosystems. Within the northwestern Arabian Gulf, *L. minuta* forms part of the characteristic neustonic copepod assemblage inhabiting warm coastal and offshore surface waters. As a surface-dwelling planktonic species, it contributes to pelagic food webs and represents an ecologically important component of Gulf zooplankton communities.

##### *Labidocera kroyeri stylifera* Thompson I.C. & Scott A., 1903

**Distribution**. Recorded from southern Iraqi marine waters in the northwestern Arabian Gulf by Khalaf (1992). The species has not been listed in Al-Yamani et al. (2011), indicating that its regional occurrence remains insufficiently documented in broader Gulf syntheses and may reflect limited sampling or taxonomic oversight.

**Remarks**. *Labidocera kroyeri stylifera* is a warm-water calanoid copepod of the family Pontellidae, characteristic of coastal and neritic marine plankton in tropical and subtropical regions. Although detailed species-specific studies are limited, members of the genus *Labidocera* are consistently reported as stable components of coastal and reef-associated copepod assemblages, often persisting across seasons and environmental gradients (Cházaro-Olvera et al. 2019). They are typically associated with nearshore waters influenced by temperature and salinity variability and can contribute to structuring plankton communities in coastal ecosystems. The Iraqi record attributed to *L. k. stylifera* by Khalaf (1992) requires critical reassessment. Subsequent regional syntheses of Arabian Gulf copepods (Al-Yamani et al. 2011; Prusova and Al-Yamani 2014) did not report this species and instead documented other representatives of the genus, including *L. bengalensis*, *L. acuta*, *L. kroyeri*, and *L. minuta*. Given the morphological similarity among several Indo–West Pacific species of *Labidocera* and the reliance on classical identification methods in earlier studies, the Iraqi record may represent a misidentification of a closely related regional taxon. Accordingly, the occurrence of *L. k. stylifera* in Iraqi waters should be regarded as requiring confirmation through re-examination of voucher material and application of modern morphological and molecular approaches.

##### *Labidocera bengalensis* Krishnaswamy, 1952

**Distribution**. Recorded from southern Iraqi marine waters in the northwestern Arabian Gulf by Khalaf (1992) and subsequently listed in regional syntheses of Gulf zooplankton, including Al-Yamani et al. (2011), confirming its occurrence within Arabian Gulf plankton communities.

**Remarks**. *Labidocera bengalensis* is a warm-water calanoid copepod of the family Pontellidae, originally described from coastal plankton off the Madras (Chennai) coast, India (Krishnaswamy 1952). It is characteristic of tropical Indo–West Pacific marine waters and typically occurs in coastal and neritic surface plankton. The species is identifiable by its relatively robust body form, distinct pigmentation, and diagnostic fifth leg morphology characteristic of the genus *Labidocera*. The species is widely distributed throughout tropical Indo–West Pacific coastal waters and forms part of the characteristic neustonic and near-surface plankton assemblages of warm marine environments. Its occurrence in Iraqi waters is consistent with its known distribution within the Arabian Gulf and broader Indo–West Pacific region. The records from Khalaf (1992) and Al-Yamani et al. (2011) indicate that *L. bengalensis* is an established component of the northwestern Arabian Gulf copepod fauna. As a surface-dwelling marine copepod, it contributes to pelagic food webs and represents part of the characteristic tropical plankton community of Iraqi coastal waters.

#### Family Temoridae Giesbrecht, 1893

##### *Temora turbinata* (Dana, 1849)

**Distribution**. Recorded from estuarine waters of southern Iraq near the Shatt Al-Arab estuary (Jebir et al. 2025) and also included in the northwestern Arabian Gulf zooplankton fauna documented by Al-Yamani et al. (2011), confirming its regular occurrence in Gulf waters adjacent to Iraq.

**Remarks**. *Temora turbinata* is a cosmopolitan warm-water marine calanoid species widely distributed in tropical and subtropical coastal waters worldwide (Campos et al. 2017; Parthasarathi et al. 2024). The species is commonly associated with coastal, shelf, and estuarine environments and is recognized as a characteristic component of warm-water plankton communities in tropical and subtropical seas. Its occurrence in both Iraqi estuarine waters and the broader northwestern Arabian Gulf is consistent with its broad ecological tolerance and widespread distribution in coastal marine ecosystems. Within the Arabian Gulf, *T. turbinata* forms part of the characteristic pelagic copepod assemblage inhabiting productive coastal and estuarine habitats. As an abundant calanoid copepod, it contributes significantly to secondary production and serves as an important trophic link between primary producers and higher consumers, including larval fishes and planktivorous invertebrates.

#### Family Tortanidae Sars G.O., 1902

##### Tortanus (Tortanus) forcipatus (Giesbrecht, 1889)

**Distribution**. Recorded from southern Iraqi marine waters in the northwestern Arabian Gulf, where Morad et al. (2013) documented the species among the copepod fauna of Iraqi coastal plankton assemblages. It is also included in Al-Yamani et al. (2011), confirming its occurrence in Arabian Gulf plankton communities.

**Remarks**. *Tortanus forcipatus* is a warm-water marine calanoid copepod of the family Tortanidae, widely distributed in Indo-Pacific coastal and neritic waters. It is a characteristic carnivorous plankton species, preying actively on smaller copepods and nauplii rather than filtering phytoplankton (Uye and Kayano 1994). A notable ecological trait is its production of long-lived resting eggs, which accumulate in sediments and allow seasonal re-establishment of planktonic populations under favorable conditions (Kasahara and Uye 1979; Dahms et al. 2006). As a predatory copepod, *T. forcipatus* occupies a relatively high trophic position within marine zooplankton communities and may influence the abundance and composition of smaller copepod assemblages. Its occurrence in Iraqi marine waters is consistent with its broad distribution throughout tropical and subtropical Indo–West Pacific coastal ecosystems. The records from the northwestern Arabian Gulf indicate that the species forms part of the characteristic marine plankton assemblage of Iraqi coastal waters. The presence of resting egg banks may facilitate population persistence in the environmentally variable conditions characteristic of shallow Gulf ecosystems.

### Iraqi cyclopoid copepods

#### Family Corycaeidae Dana, 1846

**Corycaeus (Ditrichocorycaeus) dahli Tanaka, 1957**

**Distribution**. Recorded from southern Iraqi marine waters in the northwestern Arabian Gulf, where Morad et al. (2013) documented the species among the copepod fauna of Iraqi coastal plankton assemblages. The species is also listed in Al-Yamani et al. (2011), confirming its established occurrence in Arabian Gulf plankton communities.

**Remarks**. Corycaeus (Ditrichocorycaeus) dahli is a small warm-water cyclopoid copepod of the family Corycaeidae, characteristic of tropical and subtropical marine plankton. It belongs to the subgenus *Ditrichocorycaeus* and is distinguished from related species such as *C. lubbocki* and *C. subtilis* by detailed ornamentation of the genital double somite, caudal seta proportions, and leg spine morphology (Wi et al. 2013). The species is known from Japanese and Korean waters and has also been reported from the northern Arabian Sea, including Pakistan coastal waters (Ara and Farooq 2013). Its occurrence in Iraqi marine waters is consistent with its known distribution in warm Indo–West Pacific coastal ecosystems. Within the northwestern Arabian Gulf, *C. dahli* forms part of the characteristic marine microzooplankton assemblage inhabiting coastal and offshore waters. As a small predatory cyclopoid copepod, it contributes to pelagic food webs and represents an important component of marine zooplankton biodiversity in Iraqi coastal waters.

##### *Ditrichocorycaeus lubbocki* (Giesbrecht, 1891)

**Distribution**. Recorded from southern Iraqi marine waters in the northwestern Arabian Gulf, where Morad et al. (2013) documented the species among the copepod fauna of Iraqi coastal plankton assemblages. The species is also listed in Al-Yamani et al. (2011), confirming its occurrence in Arabian Gulf plankton communities.

**Remarks**. *Ditrichocorycaeus lubbocki* is a small warm-water marine cyclopoid copepod of the family Corycaeidae. It is distinguished from related species by fine morphological traits including ornamentation of the genital double somite, relative spine lengths on swimming legs, caudal seta proportions, and detailed mouthpart structure (Wi et al. 2013). This species is characteristic of tropical and subtropical marine plankton and is widely distributed in Indo-Pacific coastal waters. Its occurrence in Iraqi marine waters is consistent with its broad distribution throughout tropical and subtropical Indo–West Pacific ecosystems. Within the northwestern Arabian Gulf, *D. lubbocki* forms part of the characteristic marine plankton assemblage inhabiting coastal and offshore waters. As a member of the Corycaeidae, it contributes to pelagic food webs and represents an important component of marine zooplankton biodiversity in Iraqi coastal waters.

##### *Corycaeus andrewsi* (Farran, 1911)

**Distribution**. Recorded from southern Iraqi marine waters in the northwestern Arabian Gulf, where Morad et al. (2013) documented the species among the copepod fauna of Iraqi coastal plankton assemblages. The species is also listed in Al-Yamani et al. (2011), confirming its established occurrence in Arabian Gulf plankton communities.

**Remarks**. *Corycaeus andrewsi* is a small warm-water marine cyclopoid copepod of the family Corycaeidae, commonly placed in the subgenus *Ditrichocorycaeus*. It is a characteristic stenohaline marine species associated mainly with high-salinity coastal and offshore waters of the Indo-Pacific, including Southeast Asia, the Bay of Bengal, and tropical oceanic regions (Mulyadi 2003; Jerez-Guerrero et al. 2022). The species is often among the numerically dominant small copepods in tropical epipelagic plankton communities, where it contributes significantly to mesozooplankton abundance and serves as prey for higher trophic levels (Jerez-Guerrero et al. 2020; Beaven et al. 2024). Its occurrence in Iraqi marine waters is consistent with its broad distribution throughout tropical Indo–Pacific marine ecosystems. Within the northwestern Arabian Gulf, *C. andrewsi* forms part of the characteristic offshore and coastal plankton assemblage associated with warm, saline marine waters. As a common epipelagic copepod, it contributes to secondary production and serves as an important trophic link between microbial and planktonic production and higher consumers in Gulf pelagic food webs.

#### Family Cyclopidae Rafinesque, 1815

##### *Cyclops vicinus vicinus* Uljanin, 1875

**Distribution**. Recorded from Iraqi freshwater rivers, marshes, and inland limnetic habitats, especially in Tigris–Euphrates riverine plankton and marsh waters (Gurney 1921; Muften et al. 2002; Al-Doori 2009).

**Remarks**. *Cyclops vicinus* is a widespread Palearctic freshwater planktonic cyclopoid and one of the characteristic limnetic cyclopoids of temperate Eurasia. Molecular–morphological revision confirms it as a valid species within a complex of approximately 15 related lineages (Krajíček et al. 2016). The occurrence of this species in Iraq is biogeographically noteworthy because it represents part of the southeastern distributional range of a predominantly temperate Eurasian freshwater lineage. Iraqi populations represent a typical native freshwater Palearctic element in rivers and marshes. In southern Iraq, the species is primarily associated with permanent freshwater habitats, including marshes and riverine systems (Gurney 1921; Muften et al. 2002; Al-Doori 2009), where it contributes to the characteristic limnetic zooplankton assemblage of the Tigris–Euphrates basin.

##### *Diacyclops bicuspidatus* (Claus, 1857)

**Distribution**. Recorded from the Tigris floodplain wetlands, Iraq (Gurney 1921).

**Remarks**. *Diacyclops bicuspidatus* belongs to the *D. bicuspidatus* species complex as defined by Karanovic and Krajíček (2012), which was recently revised using morphometric and molecular evidence, resulting in the recognition of fewer valid taxa through the synonymization of several former subspecies (Novikov et al. 2024; Smolska et al. 2024). The complex, as defined by Karanovic and Krajíček (2012), has a broad Holarctic distribution and includes several morphologically similar freshwater taxa that were historically difficult to distinguish, a pattern confirmed by recent integrative taxonomic studies (Novikov et al. 2024; Smolska et al. 2024). The Iraqi record is based on Gurney (1921), who reported the species from freshwater habitats of the Tigris–Euphrates basin. The occurrence of *D. bicuspidatus* in Iraq is consistent with the broad western Palearctic distribution of the complex and its known association with littoral, benthic, and shallow-water habitats. However, given recent taxonomic revisions within the complex, confirmation of the identity of Iraqi populations using modern morphological and molecular approaches would be desirable.

##### *Diacyclops languidoides* (Lilljeborg, 1901)

**Distribution**. Found in Iraqi freshwater small water bodies, littoral margins, and interstitial habitats.

**Remarks**. A member of the morphologically subtle *Diacyclops languidoides* complex, as defined by Karanovic et al. (2013), requiring fine ornamentation characters and detailed examination of appendage morphology for reliable identification (Novikov et al. 2024). The species complex is widely distributed throughout the western Palearctic and occupies a broad range of freshwater habitats, particularly littoral zones, small water bodies, and interstitial environments (Pesce and Galassi 1987; Rundle 1990; Reid et al. 1991). Iraqi occurrences are consistent with Palearctic freshwater distribution patterns. Its presence in northern Iraq extends the known occurrence of this Palearctic freshwater lineage into the Tigris basin and contributes to the diversity of cyclopoid copepods inhabiting Iraqi inland waters.

##### *Eucyclops agilis agilis* (Koch, 1838)

**Distribution**. Recorded from the Amara region wetlands and other freshwater habitats of the Tigris–Euphrates Basin (Gurney 1921; Al-Laami et al. 1998; Muften et al. 2002; Matlob 2004; Al-Sodani et al. 2007).

**Remarks**. *Eucyclops agilis agilis* belongs to the morphologically challenging *E. serrulatus agilis* species complex, as defined by Alekseev and Defaye (2011) and subsequently revised by Sukhikh and Alekseev (2015). Species within this complex require detailed examination of fine morphological characters, morphometric traits, and, increasingly, molecular markers for reliable identification because traditional diagnostic characters alone are often insufficient (Alekseev et al. 2006; Sukhikh and Alekseev 2015; Mercado-Salas and Suárez-Morales 2021). Members of the complex are widespread throughout the Palearctic and typically inhabit littoral and benthic freshwater environments, including ponds, lakes, marshes, springs, and shallow water bodies (Hamrová et al. 2012; Karpowicz et al. 2021). The Iraqi record is consistent with the known Palearctic distribution and ecological preferences of the complex and contributes to the diversity of cyclopoid copepods inhabiting Iraqi inland waters. Future integrative taxonomic studies will be necessary to confirm the identity of Iraqi populations.

##### *Macrocyclops albidus* (Jurine, 1820)

**Distribution**. Recorded from the southern Iraqi marshes and other freshwater habitats of the Tigris–Euphrates Basin, including marshes, canals, and vegetated inland waters (Gurney 1921; Matlob 2004; Abdulwahab and Rabee 2015).

**Remarks**. *Macrocyclops albidus* is a widespread predatory cyclopoid with a broad Holarctic distribution and has been introduced into other regions through natural dispersal and human-mediated transport (Karanovic and Krajíček 2012). The species is commonly associated with shallow vegetated freshwater habitats, including marsh margins, ponds, canals, ditches, and floodplain wetlands, where it functions as an important predator within freshwater zooplankton communities (Daggett and Davis 1974; Lansac-Tôha et al. 2002). Molecular studies indicate that *M. albidus* represents a complex of cryptic lineages, highlighting the need for integrative taxonomic approaches combining detailed morphology with molecular data (Karanovic and Krajíček 2012). In Iraq, it has been recorded from the southern marshes and other freshwater habitats of the Tigris–Euphrates Basin (Gurney 1921; Matlob 2004; Abdulwahab and Rabee 2015), consistent with its characteristic preference for vegetated littoral environments.

##### *Megacyclops viridis viridis* (Jurine, 1820)

**Distribution**. Found in shallow vegetated freshwater pools, ponds, and marsh margins in Iraq (Gurney 1921).

**Remarks**. *Megacyclops viridis viridis* is a large predatory cyclopoid widely distributed throughout the Palearctic region and commonly associated with shallow vegetated freshwater habitats, including ponds, marshes, ditches, and slow-flowing waters. The species is an important predator of small zooplankton and aquatic invertebrates and is frequently a characteristic component of littoral freshwater communities. The Iraqi record is based on Gurney (1921), who reported the species from freshwater habitats of the Tigris–Euphrates basin. Its occurrence in Iraq is consistent with the broad Palearctic distribution of the species and with the availability of suitable vegetated littoral habitats in Mesopotamian wetlands. Additional contemporary surveys would be valuable to confirm its current distribution and ecological status in Iraqi inland waters.

##### *Mesocyclops leuckarti* (Claus, 1857)

**Distribution**. Recorded from Iraqi rivers, marshes, reservoirs, and freshwater lakes (Gurney 1921).

**Remarks**. *Mesocyclops leuckarti* is the type species of the genus *Mesocyclops* and one of the most characteristic freshwater cyclopoids of the Palearctic region. Recent integrative taxonomic revisions have demonstrated that many historical records from Africa, Asia, and other regions were based on misidentifications, resulting in a more restricted Palearctic distribution for *M. leuckarti* sensu stricto (Míč et al. 2025; Selis et al. 2025).

The Iraqi record is based on Gurney (1921), who reported the species from freshwater habitats of the Tigris–Euphrates basin. Its occurrence in Iraq is consistent with the currently recognized Palearctic distribution of *M. leuckarti*. However, in light of recent taxonomic revisions within the genus, confirmation of Iraqi populations using modern morphological and molecular approaches would be valuable. Such studies would help clarify the distribution and taxonomic status of *Mesocyclops* species in Mesopotamian inland waters.

##### *Microcyclops diaphanus* (Fischer, 1853)

**Distribution**. Reported from freshwater littoral and benthic habitats in southern Iraq (Gurney 1921).

**Remarks**. The Iraqi record of *Microcyclops diaphanus* is based on a historical report from southern freshwater habitats and should be regarded as provisional pending taxonomic verification. The taxonomy of *Microcyclops* has undergone substantial revision, with recent studies demonstrating that reliable species identification requires detailed examination of fine morphological characters, including integumental ornamentation, appendage microstructure, and caudal setation, supplemented where possible by molecular data (Gutiérrez-Aguirre and Cervantes-Martínez 2016, 2023). Consequently, many historical records of widespread nominal species require re-evaluation using modern taxonomic standards (Dole-Olivier et al. 2000; Benovics et al. 2023). Re-examination of Iraqi material using integrative taxonomic approaches will be necessary to confirm the occurrence of *M. diaphanus* in the Tigris–Euphrates Basin.

##### *Apocyclops dimorphus* (Kiefer, 1934)

**Distribution**. Reported from the Diyala River and its branches in central Iraq (Al-Doori 2009).

**Remarks**. *Apocyclops dimorphus* is a euryhaline cyclopoid copepod inhabiting freshwater and brackish environments. The species was originally described from the saline waters of the Salton Sea, California, and has subsequently been reported from a variety of inland and coastal habitats, including temporary and permanent brackish water bodies in North America (Johnson 1953; Reid and Hribar 2006). Its tolerance of warm climates and elevated salinity makes its occurrence in Iraqi riverine and wetland habitats biogeographically plausible. Nevertheless, because the Iraqi record is based on a single ecological survey using older identification keys (Al-Doori 2009), additional taxonomic confirmation would be desirable.

##### *Apocyclops dengizicus dengizicus* (Lepeshkin, 1900)

**Distribution**. Recorded from the Tigris–Euphrates basin, including Garmat Ali and the Shatt Al-Arab in southern Iraq. The species was first reported by Gurney (1921) and was subsequently investigated in ecological, physiological, and life-history studies at Garmat Ali (Mohamed et al. 2004, 2008).

**Remarks**. *Apocyclops dengizicus dengizicus* is a euryhaline cyclopoid copepod widely distributed across Eurasia, North Africa, the Middle East, and Australia. The species inhabits freshwater, brackish, and saline inland waters and exhibits remarkable tolerance to environmental stress, including elevated salinity and temperature (Dexter 1993; Farhadian et al. 2014; Anufriieva 2016). Laboratory studies have demonstrated successful reproduction across a broad salinity range and optimal population growth under warm conditions, reflecting adaptation to arid and semi-arid environments (Dexter 1993; Farhadian et al. 2014). In Iraq, the species was first recorded by Gurney (1921) from the Tigris–Euphrates basin and has subsequently been reported from Garmat Ali and the Shatt Al-Arab, where local populations have been investigated in detail, including studies of life-history traits, seasonal dynamics, and oxygen consumption (Mohamed et al. 2004, 2008). These studies indicate that *A. dengizicus dengizicus* is an established component of the warm-water cyclopoid fauna of southern Iraq.

##### *Paracyclops fimbriatus fimbriatus* (Fischer, 1853)

**Distribution**. Recorded from freshwater habitats of Iraq, including the Tigris River at Baghdad and Habbaniya Lake, where it was reported by Muften et al. (2002) and Abdulwahab and Rabee (2015).

**Remarks**. *Paracyclops fimbriatus fimbriatus* is a widespread freshwater cyclopoid copepod distributed throughout Europe, North Africa, the Middle East, and Asia. The species inhabits a broad range of freshwater environments, including rivers, lakes, reservoirs, ponds, marshes, springs, and groundwater habitats (Karaytuğ et al. 1998; Mykitchak 2018). Records from Turkey and other parts of the Middle East demonstrate its occurrence in western Asia and support its presence in the Tigris–Euphrates basin (Başak et al. 2014; Can and Bozkurt 2023). Owing to its broad ecological tolerance and extensive geographic distribution, the occurrence of *P. fimbriatus* in Iraq is biogeographically expected and does not represent an unusual record. Nevertheless, species of *Paracyclops* are morphologically similar and require careful examination of fine diagnostic characters for reliable identification, particularly to distinguish *P. fimbriatus* from closely related species such as *P. imminutus* (Karaytug, 1999).

##### *Paracyclops affinis* (Sars G.O., 1863)

**Distribution**. Recorded from the southern Iraqi marshes and other freshwater habitats of southern Iraq (Gurney 1921; Matlob 2004; Al-Doori 2009; Abdulwahab and Rabee 2015).

**Remarks**. *Paracyclops affinis* is a widespread freshwater cyclopoid inhabiting ponds, lakes, marshes, canals, and other shallow freshwater environments. In Iraq, it has been recorded from the southern marshes and associated freshwater habitats (Gurney 1921; Matlob 2004; Al-Doori 2009; Abdulwahab and Rabee 2015). As with other members of the genus, reliable identification requires careful examination of fine morphological characters because closely related *Paracyclops* species may be difficult to distinguish using traditional diagnostic features alone (Karaytuğ 1999).

##### Microcyclops (Microcyclops) varicans (Sars G.O., 1863)

**Distribution**. Recorded from freshwater habitats of the Euphrates Basin, including Karbala and the Garma marshes (Al-Saboonchi et al. 1986; Hussain 2019).

**Remarks**. Microcyclops (Microcyclops) varicans was historically regarded as a cosmopolitan freshwater cyclopoid, but recent taxonomic revisions have shown that many extra-Palearctic records probably represent distinct regional species rather than *M. varicans* sensu stricto (Gutiérrez-Aguirre and Cervantes-Martínez 2016, 2023). A modern redescription restricted the confirmed distribution of *M. varicans* sensu stricto largely to the western Palearctic, based on detailed morphological characters (Mirabdullayev and Defaye 2022). In Iraq, the species has been recorded from the Euphrates Basin, including Karbala and the Garma marshes (Al-Saboonchi et al. 1986; Hussain 2019). Given the taxonomic complexity of the genus and the recognition of cryptic diversity in widespread freshwater copepods, re-examination of Iraqi material using integrative morphological and molecular approaches is desirable to confirm its identity (Gutiérrez-Aguirre and Cervantes-Martínez 2023).

##### *Thermocyclops crassus* (Fischer, 1853)

**Distribution**. Recorded from the Tigris River Basin and the Garma marshes in Iraq (Gurney 1921; Al-Saboonchi et al. 1986).

**Remarks**. *Thermocyclops crassus* is a widespread thermophilic freshwater cyclopoid occurring throughout the Palearctic, Africa, and parts of Asia, with introduced populations reported from the Americas (Fernando and Ponyi 1981; Gutiérrez-Aguirre and Suárez-Morales 2000; Campbell et al. 2024). The species typically inhabits lakes, reservoirs, marshes, rivers, and other eutrophic or slow-flowing freshwater habitats, where its abundance is strongly influenced by temperature (Maier 1989, 1996; Nowakowski and Sługocki 2024). In Iraq, it has been recorded from the Tigris River Basin and the Garma marshes (Gurney 1921; Al-Saboonchi et al. 1986). Its occurrence is consistent with its preference for warm freshwater environments, although re-examination of Iraqi material using modern taxonomic approaches would be valuable given the morphological similarity among congeners.

##### *Ectocyclops phaleratus* (Koch, 1838)

**Distribution**. Reported from Iraqi freshwater habitats by Majeed et al. (2021) and Nashaat and Al-Bahathy (2022).

**Remarks**. *Ectocyclops phaleratus* is a widespread freshwater cyclopoid copepod reported from North America, Africa, Asia, and the Middle East (Battish 1984; Dussart and Fernando 1990; Loftus and Reid 2000; Shamseldean et al. 2023). The species can generally be identified using established morphological characters, although the genus has a history of nomenclatural confusion and occasional misidentifications (Dussart and Fernando 1990; Shamseldean et al. 2023). The Iraqi records are based on recent faunistic surveys (Majeed et al. 2021; Nashaat and Al-Bahathy 2022). Nevertheless, examination of voucher material using modern morphological criteria, and where possible molecular data, would further confirm the identity of Iraqi populations.

#### Family Oithonidae Dana, 1853–1855

##### *Limnoithona tetraspina* Zhang & Li, 1976

**Distribution**. Recorded from lower Shatt Al-Arab estuarine waters of southern Iraq (Mohammed et al. 2014).

**Remarks**. *Limnoithona tetraspina* is a small estuarine cyclopoid copepod native to East Asia and widely recognized as a successful aquatic invader in brackish-water ecosystems outside its native range. The species has established invasive populations in several estuarine systems through shipping-related transport, particularly ballast water (Cordell et al. 2008; Winder and Jassby 2011).

Its occurrence in the lower Shatt Al-Arab estuary represents a notable range expansion beyond its native East Asian distribution. The species is absent from earlier syntheses of the northwestern Arabian Gulf copepod fauna (Al-Yamani et al. 2011), suggesting a relatively recent arrival or previous under-detection in the region. Given its known invasion history elsewhere and its occurrence in a major shipping corridor, *L. tetraspina* is best regarded as a probable non-native species in Iraqi waters pending further regional surveys. Continued monitoring is needed to determine its current distribution, establishment status, and potential ecological effects on native estuarine zooplankton communities.

##### *Oithona attenuata* Farran, 1913

**Distribution**. Recorded from the northwestern Arabian Gulf, Iraq (Morad et al. 2013).

**Remarks**. *Oithona attenuata* is a widespread warm-water marine planktonic copepod occurring in tropical and subtropical coastal waters of the Indo-West Pacific. It is commonly associated with neritic and estuarine environments, where it can be an abundant component of the mesozooplankton community (Hwang et al. 2010). Like other species of *Oithona*, it is an omnivorous feeder that primarily consumes ciliates and dinoflagellates and plays an important role in transferring energy from the microbial loop to higher trophic levels (Zamora-Terol et al. 2014; Wang et al. 2017). In Iraq, it has been recorded from the northwestern Arabian Gulf (Morad et al. 2013), where its occurrence is consistent with the warm, marine conditions of the Gulf.

### Iraqi harpacticoid copepods

#### Family Canthocamptidae Brady, 1880

**Canthocamptus (Canthocamptus) staphylinus staphylinus Jurine, 1820**

**Distribution**. Recorded from marsh habitats of the Tigris region in Iraq, where it was originally reported by Gurney (1921) as a native freshwater harpacticoid species. It is associated with inland freshwater marshes and shallow vegetated wetlands of southern Iraq.

**Remarks**. *Canthocamptus staphylinus* is the type species of the genus *Canthocamptus* and represents a widespread Palearctic freshwater harpacticoid distributed across Eurasian inland waters (Novikov and Sharafutdinova 2022). A recent revision of the genus restricted *Canthocamptus* to taxa closely related to *C. staphylinus*, removing many historically misassigned species (Novikov and Sharafutdinova 2022). The species exhibits marked morphological variability among populations, especially in body size, caudal ramus proportions, female P5 exopod spine lengths, and anal operculum spinulation (Kochanova and Fefilova 2017; Kochanova et al. 2018). Molecular studies based on mitochondrial COI reveal deep phylogeographic divergence among European populations, suggesting that *C. staphylinus* may represent a cryptic species complex rather than a single homogeneous taxon (Kochanova et al. 2021). Ecologically, it is a cold-adapted stenothermal freshwater species inhabiting springs, ponds, marshes, littoral lake zones, and slow-flowing rivers, where adults may form dormant cyst-like stages in bottom sediments during unfavorable periods (Kurashov 1996; Kochanova et al. 2018, 2021). The Iraqi record reported by Gurney (1921) is consistent with the broad Palearctic distribution of the species. However, given recent taxonomic revisions and evidence of cryptic diversity within the *C. staphylinus* complex, confirmation of Iraqi populations using modern morphological and molecular approaches would be desirable.

#### Family Miraciidae Dana, 1846

##### *Macrosetella gracilis* (Dana, 1848)

**Distribution**. Recorded from southern Iraqi marine waters in the northwestern Arabian Gulf, where Morad et al. (2013) documented the species among the copepod fauna of Iraqi coastal plankton assemblages. The species is also listed in Al-Yamani et al. (2011), confirming its occurrence in Arabian Gulf marine plankton communities.

**Remarks**. *Macrosetella gracilis* is a distinctive pelagic harpacticoid copepod of the family Miraciidae notable for its close ecological association with floating colonies of the cyanobacterium *Trichodesmium*. Unlike most harpacticoids, it has evolved a fully pelagic lifestyle and uses *Trichodesmium* colonies both as habitat and food source, particularly in warm oligotrophic tropical seas (Böttger-Schnack and Schnack 1989; Eberl and Carpenter 2007). The species plays an important trophic role by transferring carbon and nitrogen fixed by cyanobacteria into higher marine food webs (O’Neil and Roman 1994). Its occurrence in both Morad et al. (2013) and Al-Yamani et al. (2011) indicates that it is a native Arabian Gulf marine species naturally present in Iraqi offshore plankton communities, likely associated with seasonal cyanobacterial bloom dynamics.

##### *Delavalia longifurca* (Sewell, 1934)

**Distribution**. Recorded from southern Iraqi marshes and the Shatt Al-Arab River, including Al-Burga Marsh, Al-Qurna, and adjacent estuarine marsh habitats in Basrah Province (Mohammed 2018). The species occurs in brackish marsh–estuarine environments connected to the northern Arabian Gulf and represents one of the few documented marine-derived harpacticoids in Iraqi inland transitional waters.

**Remarks**. *Delavalia longifurca* is a marine harpacticoid copepod of the family Miraciidae, a cosmopolitan sediment-associated family widely distributed in marine and estuarine meiofaunal systems (Gómez 2021; Karanovic 2023; Tran et al. 2025). Within Miraciidae, *Delavalia* is a stenheliine genus comprising both shallow-water and deep-sea taxa, many of which are associated with coastal benthic sediments (Sham et al. 2019; Gómez 2021). In southern Iraq, *D. longifurca* was documented from multiple marsh and estuarine localities between 2006–2009, with the highest abundance reaching 231 ind./m^3^ in Al-Burga Marsh and 92 ind./m^3^ in the Shatt Al-Arab at Al-Qurna (Mohammed 2018). These records confirm that the species is a regular native component of Iraq’s marine-influenced marsh meiofauna rather than an accidental occurrence. Iraqi specimens differ slightly from Sewell’s original Indian material in several morphological details, although they remain assignable to *D. longifurca* (Mohammed 2018), suggesting either regional morphological divergence or cryptic population-level differentiation within this broadly Indo-West Asian estuarine taxon. Its occurrence extends the known distribution of *Delavalia* into the northern Arabian Gulf–Mesopotamian estuarine corridor and highlights the underexplored diversity of harpacticoid copepods in southern Iraqi transitional ecosystems.

#### Family Tachidiidae Sars G.O., 1909

##### *Euterpina acutifrons* (Dana, 1848)

**Distribution**. Recorded from southern Iraqi marine waters in the northwestern Arabian Gulf, where Morad et al. (2013) documented the species among the copepod fauna of Iraqi coastal plankton assemblages. It is also listed in Al-Yamani et al. (2011), confirming its established occurrence in Arabian Gulf plankton communities.

**Remarks**. *Euterpina acutifrons* is a cosmopolitan pelagic harpacticoid copepod of the family Tachidiidae, widely distributed in shallow marine and estuarine waters worldwide. Unlike most harpacticoids, it is planktonic rather than benthic and is often abundant in nutrient-rich coastal systems, where it may dominate zooplankton communities and serve as an important grazer of phytoplankton (Sautour and Castel 1995; Ara 2001). The species is highly tolerant of eutrophic and environmentally stressed habitats, making it a frequent indicator species in polluted or nutrient-enriched coastal waters (Annabi-Trabelsi et al. 2022; Rosas et al. 2024). Its occurrence in Iraqi marine waters is consistent with its broad cosmopolitan distribution in tropical and subtropical coastal ecosystems. As one of the most widespread planktonic harpacticoids, *E. acutifrons* contributes to secondary production and energy transfer within marine and estuarine food webs of the northwestern Arabian Gulf.

#### Family Peltidiidae Claus, 1860

##### *Clytemnestra scutellata* Dana, 1848

**Distribution**. Recorded from southern Iraqi marine waters in the northwestern Arabian Gulf, where Morad et al. (2013) documented the species among the copepod fauna of Iraqi coastal plankton assemblages. It is also listed in Al-Yamani et al. (2011), confirming its occurrence in Arabian Gulf plankton communities.

**Remarks**. *Clytemnestra scutellata* is a small planktonic harpacticoid copepod notable for occupying the water column rather than benthic substrates, unlike most harpacticoids. It is widely distributed in warm coastal and estuarine waters and is recognized as a regular component of Indo-Pacific marine zooplankton assemblages (Mulyadi 2010). Recent ecological studies identify it as an indicator species in eutrophic tropical estuaries, where its abundance reflects environmental gradients such as salinity and nutrient enrichment (Rosas et al. 2024). Its occurrence in Iraqi marine waters is consistent with its known distribution in tropical and subtropical Indo–Pacific coastal ecosystems. As one of the relatively few planktonic harpacticoids, *C. scutellata* contributes to marine zooplankton communities of the northwestern Arabian Gulf and reflects the ecological connectivity of Iraqi coastal waters with the broader Gulf marine ecosystem.

## Discussion

The present checklist indicates that the currently documented diversity of free-living copepods in Iraq (51 species) remains relatively modest compared with that of better-studied regions. However, regional comparisons must be interpreted cautiously because reported species richness is strongly influenced by sampling effort, spatial scale, and habitat coverage. For example, a synthesis of the Turkish part of the Euphrates–Tigris River Basin recorded only 12 copepod species at the basin level, whereas Turkish inland waters as a whole support at least 141 copepod species (Ergönül and Atasağun 2021). This discrepancy highlights how basin-scale inventories may substantially underestimate true diversity when surveys are spatially restricted.

A similar pattern is evident in Iraq. Most available studies have focused on planktonic assemblages in major rivers, reservoirs, and selected marsh systems, often based on limited temporal sampling and incomplete taxonomic resolution (Abdulwahab and Rabee 2015; Nashaat et al. 2021; Al-Keriawy and Al-Kalidy 2023). Consequently, large portions of Iraq’s aquatic environments remain underexplored, including benthic sediments, littoral zones, groundwater and hyporheic systems, temporary inland waters, and offshore sectors of the northwestern Arabian Gulf. Studies from other regions consistently demonstrate that such habitats harbor a significant proportion of copepod diversity, including cryptic and endemic taxa (Galassi et al. 2009; Stamou et al. 2022).

Another factor likely contributing to the currently underestimated diversity of Iraqi copepods is the presence of cryptic species. Molecular and integrative taxonomic studies have demonstrated that many widespread freshwater copepods previously regarded as single, broadly distributed species actually comprise complexes of genetically distinct lineages (Marrone et al. 2013; Karanovic et al. 2016; Kochanova et al. 2021). Consequently, morphology-based identifications alone may underestimate regional diversity and obscure biogeographical patterns. Integrative approaches combining detailed morphological examination with molecular data will therefore be essential for accurately delimiting species boundaries and evaluating the true diversity of Iraqi copepods.

When placed within a broader regional framework, Iraqi copepod diversity appears comparable to that of other under-sampled areas rather than genuinely species-poor systems. Inland-water inventories from Mediterranean and European regions often report higher diversity, but these typically integrate long-term, multi-habitat surveys, including subterranean and benthic environments (Stamou et al. 2022). Similarly, marine and estuarine copepod assemblages from the Levantine Basin and adjacent eastern Mediterranean systems show high diversity when shelf, coastal, and transitional habitats are comprehensively sampled (Uysal et al. 2002).

This comparison suggests that the apparent discrepancy between Iraq and neighboring regions reflects differences in research intensity rather than fundamental ecological constraints. Given its geographic position and environmental heterogeneity, Iraq likely supports a more diverse copepod fauna than is currently documented.

The geographical position of Iraq also contributes to the distinctive composition of its copepod fauna. The Tigris and Euphrates rivers originate in the Anatolian highlands, receive tributaries draining the Zagros Mountains, and ultimately discharge into the northwestern Arabian Gulf. This hydrological continuity links freshwater ecosystems across southeastern Türkiye, western Iran, Syria, and Iraq, providing opportunities for faunal exchange among adjacent drainage systems. Similar biogeographical relationships have been recognized for other freshwater organisms in the region, including fishes and freshwater mussels, highlighting the role of the Tigris–Euphrates Basin as an important corridor connecting western Palearctic and southwestern Asian freshwater faunas (Coad and Vilenkin 2004; Lopes-Lima et al. 2021; Bayçelebi et al. 2024).

A particularly noteworthy aspect of the Iraqi copepod fauna is the occurrence of several taxa generally associated with temperate or western Palearctic freshwater assemblages. Species such as *Eudiaptomus vulgaris*, and *Cyclops vicinus* are characteristic elements of temperate Eurasian faunas and are more commonly reported from cooler regions of Europe and western Asia (Boxshall and Defaye 2007; Stamou et al. 2022). Their occurrence in Iraqi inland waters is therefore biogeographically significant and extends the known distribution of several temperate freshwater taxa into southern Mesopotamia. The occurrence of these taxa in the Tigris–Euphrates Basin suggests historical connections between Iraqi freshwater ecosystems and temperate western Palearctic faunas. Geological and phylogeographic studies indicate that the drainage network has experienced repeated episodes of hydrological reorganization since the Miocene, creating opportunities for dispersal and faunal exchange among neighboring basins (Khoshnamvand et al. 2025). In addition, the Mesopotamian Marshes have long represented one of the largest wetland complexes in the Middle East and may have served as important refugial habitats for freshwater organisms during periods of environmental change (Naser et al. 2025). Although these historical processes have not been investigated specifically for Iraqi copepods, they provide plausible explanations for the occurrence of temperate freshwater taxa in southern Mesopotamia. Marine and estuarine copepods recorded from the northwestern Arabian Gulf and Shatt Al-Arab estuary primarily reflect the natural environmental gradient linking freshwater, brackish, and marine habitats in southern Iraq. Rather than representing a unique biogeographic phenomenon, these taxa are consistent with patterns observed in many river–estuary systems worldwide. One of the most distinctive biogeographical patterns observed in the Iraqi fauna is the persistence of temperate Eurasian freshwater taxa within an arid subtropical region situated at the crossroads of major biogeographical realms.

The present checklist provides a foundation for future studies but also highlights important knowledge gaps in the distribution and taxonomy of Iraqi copepods. Future research should prioritize systematic surveys across underrepresented environments, including groundwater systems, temporary inland waters, benthic and meiofaunal habitats, and offshore marine zones. Integrating modern taxonomic approaches, including detailed morphological revision and molecular tools, will be essential for resolving cryptic diversity and improving species-level identification (Boxshall and Defaye 2007; Galassi et al. 2009).

Such efforts are expected to significantly expand the known fauna and provide a more accurate basis for regional and global biogeographic comparisons. As one of the least-studied regions in western Asia, Iraq represents an important gap in current knowledge of freshwater copepod diversity. A more comprehensive understanding of its fauna will not only improve national biodiversity inventories but also contribute to resolving broader questions concerning the historical biogeography, dispersal, and evolution of freshwater copepods across the Middle East.

## Conclusions

This study provides the first comprehensive, taxonomically standardized national checklist of free-living copepods in Iraq and establishes a unified baseline for understanding the country’s copepod biodiversity across freshwater, estuarine, and marine-influenced habitats. By integrating records spanning more than a century (1921–2025), it highlights historical inconsistencies in nomenclature and presents the first coherent synthesis of Iraqi copepod diversity.

The currently recognized free-living copepod fauna of Iraq comprises 51 valid species belonging to three orders: Calanoida (26 species), Cyclopoida (20 species), and Harpacticoida (5 species). Several additional literature records were considered doubtful and excluded from the accepted checklist pending taxonomic confirmation. The checklist also demonstrates that current knowledge of Iraqi copepod diversity remains incomplete because several aquatic habitats have received limited sampling and some historical records require further taxonomic verification. The results underscore Iraq’s biogeographical significance as a transition zone linking Palearctic freshwater systems with marine-influenced assemblages of the Arabian Gulf. Marine-origin taxa constitute a substantial proportion of the recorded fauna (56.9%), reflecting strong connectivity with Gulf plankton communities. The occurrence of temperate western Palearctic freshwater taxa together with marine and estuarine species highlights the importance of the Tigris–Euphrates Basin as a biogeographical corridor linking major aquatic faunas of western Asia. Future progress in Iraqi copepod research will depend on expanded nationwide sampling, re-examination of doubtful historical records, and the application of integrative taxonomic approaches combining morphology with molecular tools to improve species delimitation and clarify evolutionary relationships. Long-term monitoring across freshwater-to-marine salinity gradients will also be essential for detecting biodiversity change under increasing pressures from salinization, hydrological alteration, habitat degradation, and biological invasions, all of which are rapidly reshaping Iraq’s aquatic ecosystems.

This checklist provides a critical reference framework for future ecological, taxonomic, and biogeographical studies and forms an essential foundation for long-term biodiversity assessment, aquatic ecosystem monitoring, and conservation planning in Iraq, while also contributing to a broader understanding of freshwater biodiversity and biogeographical patterns across the Middle East.

## Additional information

### Artificial Intelligence (AI) use

The authors accept full responsibility for the content of the manuscript, including the disclosure of any use of AI.

AI tools were used for English language improvement only.

### Author ORCIDs

M. Naser https://orcid.org/0000-0001-5047-7657

A. Yasser https://orcid.org/0000-0002-2318-5779

M. Karpowicz https://orcid.org/0000-0003-0711-5903

P.R. de Los Ríos-Escalante https://orcid.org/0000-0001-5056-7003

### Data availability

All of the data that support the findings of this study are available in the main text or Supplementary Information.

## Supplementary materials

Supplementary material 1 Doubtful, erroneous, excluded, and records requiring confirmation of copepod taxa reported from Iraq Data type docx https://binary.pensoft.net/file/1776165This dataset is made available under the Open Database License (http://opendatacommons.org/licenses/odbl/1.0/). The Open Database License (ODbL) is a license agreement intended to allow users to freely share, modify, and use this Dataset while maintaining this same freedom for others, provided that the original source and author(s) are credited. Murtada Naser, Amaal Yasser, Maciej Karpowicz, Patricio R. de Los Ríos-Escalante
