# Supplementary material for: An annotated checklist of free-living Copepoda (Crustacea) of Iraq: diversity, distribution, and biogeographical affinities
Source: Zookeys. 2026 Sep 16;1292:177–225. doi: 10.3897/zookeys.1292.196683 (PMC13601922; doi:10.3897/zookeys.1292.196683)
Supplement: Supplementary file 1 [file zookeys-1292-177_article-196683__-s001.docx]

**Supplementary Material 1. Doubtful, erroneous, excluded, and records requiring confirmation of copepod taxa reported from Iraq**

| **Taxon (as reported)** | **Correct / Interpreted Name** | **Reason for Exclusion / Uncertainty** | **Reference** |
| --- | --- | --- | --- |
| *Diaptomus arapahoensis* | *Arctodiaptomus arapahoensis* (Dodds, 1915) | Nearctic species; occurrence in Iraq biogeographically implausible | Nashaat et al. (2021) |
| *Diaptomus amatitlanensis* | *Mastigodiaptomus amatitlanensis* (Wilson M.S., 1941) | Neotropical species; occurrence in Iraq biogeographically implausible | Nashaat et al. (2021) |
| *Diaptomus floridanus* | *Arctodiaptomus* *floridanus* (Marsh, 1926) | Nearctic species; likely misidentification | Nashaat et al. (2021) |
| *Diaptomus novemdecimus* | *Hesperodiaptomus*  *novemdecimus* Wilson M.S., 1953 | Nearctic species; likely misidentification | Nashaat and Al-Bahathy (2022) |
| *Diaptomus birgei* | *Onychodiaptomus birgei* (Marsh, 1894) | Nearctic species; occurrence in Iraq biogeographically implausible | Abdulwahab and Rabee (2015); Merza (2017) |
| *Diaptomus dorsalis* | *Arctodiaptomus* *dorsalis* (Marsh, 1907) | Nearctic species; no confirmed records from the Middle East | Abdulwahab and Rabee (2015) |
| *Diaptomus reighardi* | *Skistodiaptomus reighardi* (Marsh, 1895) | Nearctic species; likely misidentification | Al-Doori (2009); Merza (2017) |
| *Diaptomus dilobatus* | *Aglaodiaptomus*  *Dilobatus* Wilson M.S., 1958 | Taxonomically doubtful record; occurrence in Iraq unsupported | Al-Doori (2012); Merza (2017) |
| *Diaptomus sarsi* | *Sinodiaptomus sarsi* (Rylov, 1923) | Record requires verification; taxonomic identity and occurrence in Iraq remain uncertain | Abdulwahab and Rabee (2015); Merza (2017) |
| *Diaptomus leptopus* | *Arctodiaptomus leptopus* Nicolet, 1849 | record requiring reassessment under current taxonomy | Al-Doori (2012); Merza (2017) |
| *Diaptomus gracilis* | *Eudiaptomus gracilis* (Sars G.O., 1863) | record reported under outdated nomenclature; occurrence in Iraq requires confirmation | Majeed et al. (2021) |
| *Aglaodiaptomus forbesi* |  | Nearctic species; occurrence in Iraq biogeographically implausible | Majeed et al. (2021) |
| *Aglaodiaptomus lintoni* |  | Nearctic species; occurrence in Iraq biogeographically implausible | Majeed et al. (2021) |
| *Aglaodiaptomus marshianus* |  | Nearctic species; occurrence in Iraq biogeographically implausible | Majeed et al. (2021) |
| Sinodiaptomus valkanovi |  | Record based on a recent morphological study from Karbala; occurrence in Iraq requires independent verification and confirmation using modern taxonomic and molecular approaches. | Abdulshahed (2023) |
| *Hesperodiaptomus franciscanus* |  | Genus restricted to North America; occurrence in Iraq highly unlikely | Majeed et al. (2021); Merza (2017) |
| *Cyclops capillatus* | *Acanthocyclops capillatus* (Sars G.O., 1863) | record requiring taxonomic confirmation. | Abdulwahab and Rabee (2015) |
| *Cyclops exilis* | *Acanthocyclops exilis* (Coker, 1934) | record requiring taxonomic confirmation. | Abdulwahab and Rabee (2015) |
| *Cyclops venustoides* | *Acanthocyclops venustoides* venustoides (Coker, 1934) | record requiring taxonomic confirmation. | Majeed et al. (2021) |
| *Cyclops vernalis* | *Acanthocyclops vernalis vernalis* (Fischer, 1853) | record requiring verification; occurrence in Iraq remains uncertain because the record has not been subsequently confirmed and represents a biogeographically unexpected temperate taxon in southern Mesopotamian wetlands. | Al-Saboonchi et al. (1986); Al-Doori (2012), |
| *Cyclops strenuus* |  | Given the taxonomic complexity of the Cyclops strenuus species group and the absence of modern diagnostic verification, the record should be considered uncertain. | Al-Ameen (2013) |
| Cyclops scutifer |  | record requiring verification; the occurrence of this predominantly cold-temperate freshwater species in Iraq is biogeographically unexpected and has not been subsequently confirmed. The record requires reassessment using modern taxonomic criteria. | Al-Ameen (2013) |
| *Diacyclops crassicaudis* |  | Given the taxonomic complexity of the D. crassicaudis species group, the Iraqi record requires re-examination using modern diagnostic criteria. | Al-Doori (2012) |
| *Eucyclops macrurus* |  | Taxonomic identity uncertain; requires verification | Muften, et al. (2002) |
| *Ectocyclops speratus* | *Eucyclops (Speratocyclops) speratus speratus* (Lilljeborg, 1901) | Doubtful identification; occurrence requires confirmation | Abdulwahab and Rabee (2015) |
| *Tropocyclops prasinus prasinus* |  | **Record requiring taxonomic confirmation.** The only Iraqi record is from Abdulwahab and Rabee (2015), who did not provide the taxonomic source, identification key, diagnostic characters, illustrations, or voucher information used for species identification. Moreover, Tropocyclops prasinus is now recognized as a species complex requiring modern taxonomic revision; therefore, the Iraqi record requires confirmation before inclusion in the confirmed checklist. | Abdulwahab and Rabee (2015) |
| *Thermocyclops hyalinus* |  | record requiring taxonomic confirmation. | Majeed et al. (2021) |
| *Parastenocaris starreti* | *Parastenocaris brevipes* Kessler, 1913 | Doubtful record requiring verification. Species of Parastenocaris are primarily groundwater and interstitial harpacticoids requiring detailed morphological examination for reliable identification. The record is based on a local ecological survey and neither P. starreti nor P. brevipes has been reliably documented from Iraq or the Middle East. | Matlob (2004) |
| *Megacyclops magnus* |  | Nearctic species; occurrence in Iraq unsupported | Majeed et al. (2021) |
| *Megacyclops latipes* |  | Occurrence in Iraq requires confirmation | Majeed et al. (2021) |
| *Nitokra lacustris* |  | record requiring taxonomic confirmation. | Majeed et al. (2021) |
| *Nitokra spinipes* | *Nitocra spinipes* spinipes Boeck, 1865 | Species complex; occurrence requires confirmation | Abdulwahab and Rabee (2015); Matlob (2004). |
| *Nitocra lacucetrus* | *Nitokra lacustris* (Schmankevitsch, 1875) | Misspelling; invalid taxon name | Nashaat et al. (2021) |
| *Nitocra cacustrus* |  | Taxonomic identity uncertain; the name could not be confidently matched to any currently | Abdulwahab and Rabee (2015) |
| *Nitocra lacustris* |  | Record requiring verification; identification based on limited information and not subsequently confirmed from Iraq. | Muften et al. (2002) |
| *Pesceus reggiae* | *Paracamptus reggiae* Wilson M.S., 1958 | Pesceus is a replacement generic name that has subsequently been synonymized with Paracamptus; the record is therefore updated to the currently accepted genus. error. | Nashaat et al. (2021) |
| *Acroperus harpae* |  | Not a copepod (Cladocera) | Nashaat et al. (2021) |
| Elaphoidella grandidieri |  | record requiring verification; reported from Garma Marshes by Al-Saboonchi et al. (1986) but has not been subsequently confirmed from Iraq and requires taxonomic reassessment. | Al-Saboonchi et al. (1986) |
| *Elaphoidella sewelli* |  | record requiring verification; reported from Garma Marshes by Al-Saboonchi et al. (1986) but has not been subsequently confirmed from Iraq and requires taxonomic reassessment. | Al-Saboonchi et al. (1986) |
| *Mesochra lilljeborgii* |  | record requiring verification; reported from the Euphrates River, but the occurrence of this predominantly brackish-water harpacticoid in Iraqi freshwater habitats has not been subsequently confirmed and requires taxonomic reassessment. | Matlob (2004) |
